# Clinical Significance of Tumor Infiltrating Lymphocytes in Association with Hormone Receptor Expression Patterns in Epithelial Ovarian Cancer

**DOI:** 10.3390/ijms22115714

**Published:** 2021-05-27

**Authors:** Gwan Hee Han, Ilseon Hwang, Hanbyoul Cho, Kris Ylaya, Jung-A Choi, Hyunja Kwon, Joon-Yong Chung, Stephen M. Hewitt, Jae-Hoon Kim

**Affiliations:** 1 Department of Obstetrics and Gynecology, Yonsei University College of Medicine, Seoul 03722, Korea; lachicabona@gmail.com (G.H.H.); hanbyoul@yuhs.ac (H.C.); 2 Department of Obstetrics and Gynecology, Kyung Hee University Hospital at Gangdong, Seoul 05278, Korea; 3 Experimental Pathology Laboratory, Laboratory of Pathology, Center for Cancer Research, National Cancer Institute, National Institutes of Health, Bethesda, MD 20892, USA; ilseon.hwang@gmail.com (I.H.); ylayakri@mail.nih.gov (K.Y.); chungjo@mail.nih.gov (J.-Y.C.); genejock@helix.nih.gov (S.M.H.); 4 Dongsan Medical Center, Department of Pathology, Keimyung University School of Medicine, Daegu 42601, Korea; 5 Department of Obstetrics and Gynecology, Gangnam Severance Hospital, Yonsei University College of Medicine, Seoul 06229, Korea; jachoi@yuhs.ac (J.-A.C.); 89hjkwon@hanmail.net (H.K.); 6 Institute of Women’s Life Medical Science, Yonsei University College of Medicine, Seoul 03722, Korea

**Keywords:** epithelial ovarian cancer, hormone receptors, triple dominant group, tumor infiltrating lymphocytes, regulatory T cell

## Abstract

Hormone receptor expression patterns often correlate with infiltration of specific lymphocytes in tumors. Specifically, the presence of specific tumor-infiltrating lymphocytes (TILs) with particular hormone receptor expression is reportedly associated with breast cancer, however, this has not been revealed in epithelial ovarian cancer (EOC). Therefore, we investigated the association between hormone receptor expression and TILs in EOC. Here we found that ERα, AR, and GR expression increased in EOC, while PR was significantly reduced and ERβ expression showed a reduced trend compared to normal epithelium. Cluster analysis indicated poor disease-free survival (DFS) in AR+/GR+/PR+ subgroup (triple dominant group); while the Cox proportional-hazards model highlighted the triple dominant group as an independent prognostic factor for DFS. In addition, significant upregulation of FoxP3+ TILs, PD-1, and PD-L1 was observed in the triple dominant group compared to other groups. NanoString analyses further suggested that tumor necrosis factor (TNF) and/or NF-κB signaling pathways were activated with significant upregulation of *RELA*, *MAP3K5*, *TNFAIP3*, *BCL2L1*, *RIPK1*, *TRAF2*, *PARP1*, and *AKT1* in the triple dominant EOC group. The triple dominant subgroup correlates with poor prognosis in EOC. Moreover, the TNF and/or NF-κB signaling pathways may be responsible for hormone-mediated inhibition of the immune microenvironment.

## 1. Introduction

Epithelial ovarian cancer (EOC) is one of the most lethal gynecologic malignancies, responsible for more than 100,000 cancer-related deaths annually, worldwide [1]. The majority of patients are diagnosed at an advanced stage because of the lack of specific symptoms and effective cancer screening methods at the early stages. To date, the standard treatment for EOC is primary cytoreductive surgery followed by platinum-based chemotherapy. However, most patients experience relapse within 2 years and develop chemotherapy resistance. Emerging evidence has highlighted the potential of targeted therapies, such as anti-angiogenic therapy with bevacizumab or poly (ADP-ribose) polymerase (PARP) inhibitors. However, their contribution toward improving survival and prognosis remains modest [2]. Although current clinical trials on single-agent programmed cell death protein 1 (PD-1) blockade demonstrated promising results in EOC treatment, the overall response rate is considerably lower than that achieved in melanoma, lung cancer, and renal cell cancer [3].

Many studies have identified EOC as an immunogenic tumor, recognizable by the host immune system [4]. Therefore, evaluating the tumor-infiltrating lymphocytes (TILs) in EOC has recently gained attention [5,6]. TILs are the type of white blood cells present in the tumor islets and stroma that recognize tumor cells and elicit an immune response. In EOC, CD8^+^, CD4^+^ T-helper 1 (Th1), and natural killer (NK) cells reportedly participate in tumor suppression responses, whereas CD4^+^ T-helper 2 (Th2), FoxP3^+^ T-regulatory (Treg), and dendritic cells contribute to immunosuppression [7]. However, the activity and presence of TILs can be modulated by many factors, including endocrine and proinflammatory molecules. Estrogen receptor (ER)α influences cancer development by regulating cellular development and differentiation, whereas ERβ prevents EOC development. Accumulating evidence suggests that the androgen receptor (AR) is associated with the progression and development of EOC [8]. Moreover, at low concentrations, the progesterone receptor (PR) stimulates EOC progression, while suppressing it at high concentrations [9,10,11,12]. As nearly all immune cells express hormone receptors, and several immune-related genes possess AR and ER response elements in their promoters, hormone receptors can modulate B cell, T cell, macrophage, neutrophil, and NK cell behaviors [13,14,15,16,17]. Indeed, studies on the breast cancer tumor microenvironment (TME) suggest that hormone receptor-positive breast tumors may be immunologically “colder” than their triple-negative and human epidermal development element receptor 2 (HER2)-positive counterparts [18]. These observations suggest that hormone receptors influence the expression of genes in TILs in cancers. However, little is known regarding the expression of genes in TILs in relation to specific hormone receptor expression profiles in EOC.

EOC is a highly heterogeneous disease, and emerging evidence suggests that targeted therapy is applicable based on subgroup-specific prognostic and predictive biomarkers. Therefore, we aimed to comparatively analyze the relationships between the properties of TILs and hormone receptor expression patterns in EOC. Furthermore, we aimed to evaluate the usefulness of a subgroup-specific prognostic biomarker for immunotherapy.

## 2. Results

### 2.1. Expression of Hormone Receptors in EOC Tissues

**Figure 1:**
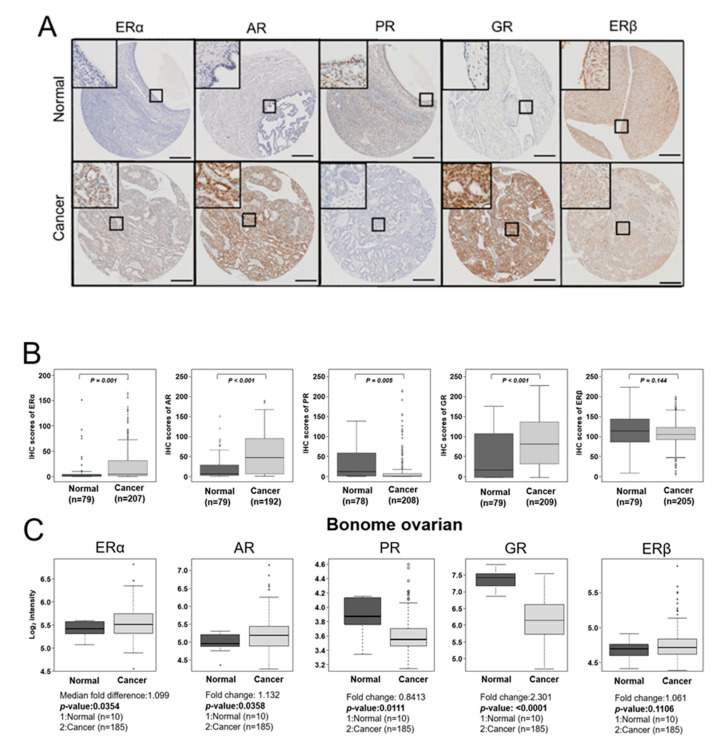

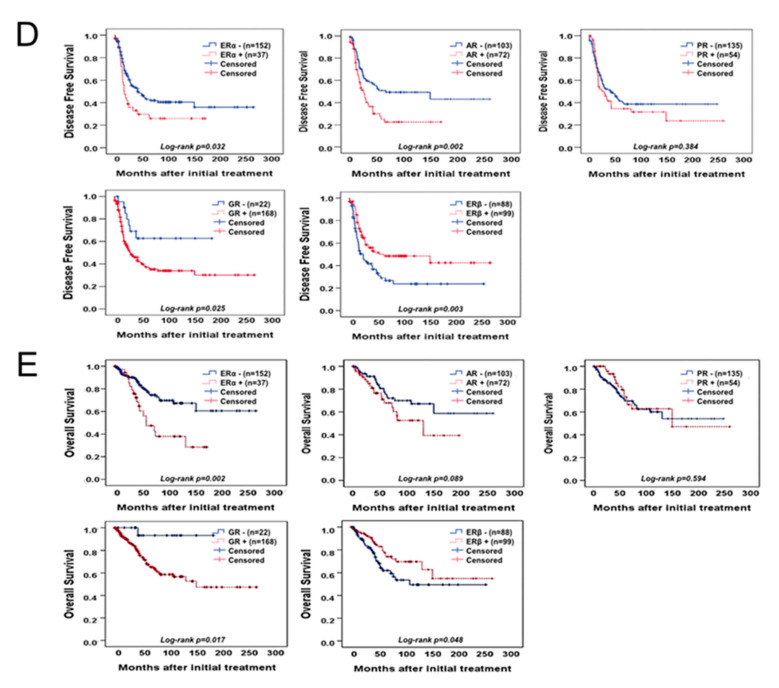
Expression of hormone receptors in EOC tissues. (**A**) Representative immunohistochemical staining images of ERα, AR, PR, GR, and ERβ in nonadjacent ovarian epithelial tissues (Normal) and epithelial ovarian cancer (Cancer) tissue samples (scale bar: 50 μm). (**B**) Boxplots of IHC staining data (histoscores) comparing between nonadjacent ovarian epithelial tissues (Normal) and epithelial ovarian cancer (Cancer) tissue samples of each hormone receptor subtype. Histoscores were calculated based on staining intensity and the area of positive staining (**C**) Publicly available data on the mRNA expression of ERα, AR, PR, GR, and ERβ were obtained from the GEO data (GSE26712). A Mann–Whitney U-test or Kruskal–Wallis test was used to compare the mRNA expression level of each hormone receptor. (**D**) The DFS of patients with EOC depends on ERα, AR, PR, GR, and ERβ expression. For the DFS analysis, 189 patients with EOC for ERα, 175 patients with EOC for AR, 189 patients with EOC for PR, 190 patients with EOC for GR, and 187 patients with EOC for ERβ were included. (**E**) The OS of patients with EOC depends on ERα, AR, PR, GR, and ERβ expression. For the OS analysis, 189 patients with EOC for ERα, 175 patients with EOC for AR, 189 patients with EOC for PR, 190 patients with EOC for GR, and 187 patients with EOC for ERβ were included. The cut-off value of ERα was over 49.2 of the IHC score, cut-off value of AR was over 10.85 of the IHC score, cut-off value of PR was over 21.18 of the IHC score, and the cut-off value of GR was over 8.65 of the IHC score.

The representative IHC results for ERα, AR, GR, PR, and ERβ are shown in Figure 1A, and IHC scores are summarized in Supplementary Table S1 and Figure 1B. The data showed significantly higher expression of ERα, AR, and GR in EOCs than in nonadjacent normal epithelial tissues (*p =* 0.001, *p* < 0.001, *p* < 0.001; respectively; Supplementary Table S1 and Figure 1B). Conversely, PR expression was significantly lower in EOCs than in nonadjacent normal epithelial tissues (*p* = 0.005; Supplementary Table S1 and Figure 1B). Although not significant, ERβ expression tended to be low in EOCs was compared to that in nonadjacent normal epithelial tissues (*p* = 0.144; Supplementary Table S1 and Figure 1B). Next, we confirmed the expression levels of ERα, AR, PR, and ERβ hormone receptors using a publicly available dataset, which showed good agreement with the trends observed in our study (Figure 1C). However, GR expression showed the opposite result, and ERβ expression did not show any significant differences, which might be due to differences in protein stability which has been extensively studied in hormone receptors [19]. We then investigated clinicopathological characteristics based on hormone receptor expression patterns. The immunoreactivity of ERα and PR was significantly associated with serous cell type (*p* = 0.027, *p* = 0.008, respectively; Supplementary Table S1), and AR overexpression significantly correlated with positive CA125 (*p* = 0.024; Supplementary Table S1). In addition, ERβ and GR overexpression were significantly associated with early (*p* = 0.047; Supplementary Table S1) and advanced FIGO stage (*p* = 0.050; Supplementary Table S1), respectively.

Next, we examined the relationship between hormone receptor expression and DFS and OS in patients with EOC using Kaplan–Meier plots. Results showed that ERα or GR overexpression correlated with poor DFS (*p* = 0.032, *p* = 0.025, respectively; Figure 1D) and OS (*p* = 0.002, *p* = 0.017, respectively; Figure 1E). On the contrary, ERβ overexpression significantly correlated with improved DFS and OS (*p* = 0.003, *p* = 0.048, respectively; Figure 1D,E). AR overexpression significantly correlated with poor DFS, whereas AR overexpression showed a statistically non-significant correlation with better OS (*p* = 0.002, *p* = 0.089, respectively; Figure 1D,E). Notably, PR expression was lower in EOCs than in nonadjacent normal epithelial tissues; however, its overexpression in EOCs showed a trend toward poor DFS (*p* = 0.384; Figure 1D).

### 2.2. Hierarchical Clustering Analysis of Hormone Receptors in EOCs

**Figure 2:**
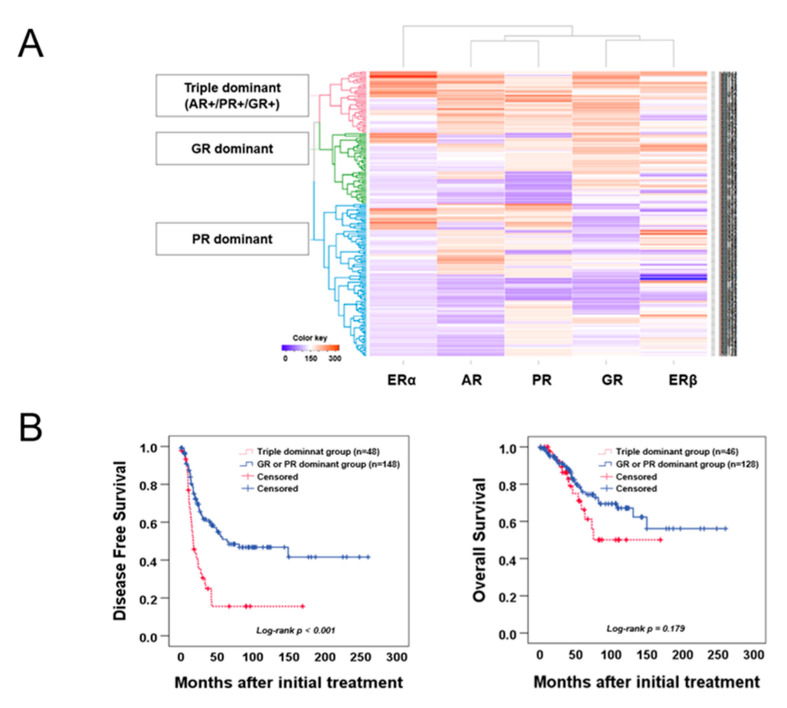

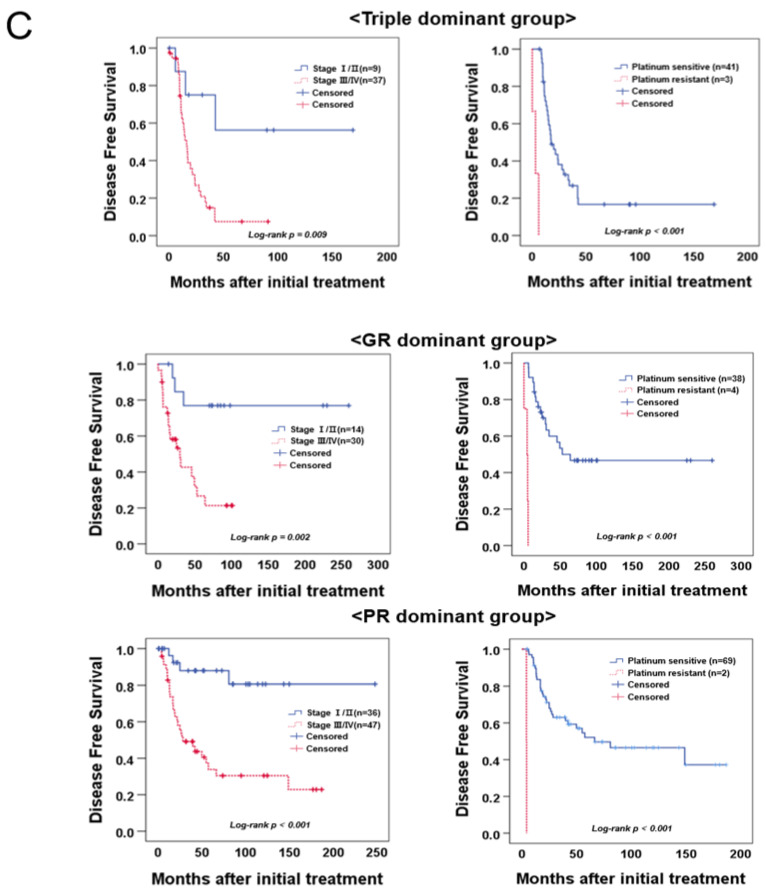
Hierarchical clustering analysis of hormone receptor expression in EOCs. (**A**) Hierarchical clustering analysis of all patient samples in which staining for all five receptors was performed. On the left, the three subgroups identified using the clustering analysis are indicated. Vertically, the expression of different receptors is depicted: orange indicating positive expression and purple indicating negative expression. Color scaling was based on the semi-quantitative immunoscores. (**B**) DFS and OS of patients with EOC between the triple dominant group and GR- or PR-dominant group. Forty-eight patients with EOC in the triple dominant group and 148 patients with EOC in the GR- or PR-dominant group were included. (**C**) Subgroup DFS analysis in the triple dominant group was based on the FIGO stage and platinum sensitivity (n = 196).

EOC was categorized into three different subgroups: triple dominant (AR+/PR+/GR+), GR-dominant, and PR-dominant, using hierarchical clustering analysis, which was performed to identify subgroups based on hormone receptor expression patterns related to prognosis after evaluating the clinicopathological characteristics based on a single hormone receptor (Figure 2A and Supplementary Figure S1A). The triple dominant group was significantly associated with a more advanced FIGO stage and serous cell type and poor grade compared with GR- or PR-dominant groups (*p* = 0.015, *p* = 0.042, *p* = 0.042, respectively; Supplementary Figure S1B). Moreover, we observed that the triple dominant group was significantly associated with poor DFS (*p* < 0.001; Figure 2B). Cox multivariate proportional-hazards analysis further revealed that the triple dominant group was an independent prognostic factor for poor DFS (HR = 2.176, 95% CI = 1.361–3.487; *p* = 0.001; Table 1). Notably, only the triple dominant group showed statistical significance in Cox multivariate proportional-hazards analysis compared with the groups with single dominant hormone receptors (Table 1). Moreover, we performed separate subgroup analyses in the triple, GR-, and PR-dominant groups (Figure 2C). Survival analysis showed that triple, GR-, and PR-dominant groups were significantly associated with poor DFS in patients at a more advanced stage, who developed platinum resistance (*p* = 0.009, *p* < 0.001, *p* = 0.002, *p* < 0.001, *p* < 0.001, *p* < 0.001, respectively; Figure 2C). The subgroup analysis results with respect to cell type, grade, and positive CA125 are presented in Supplementary Figure S2.

### 2.3. Association between TIL Infiltration and EOC Subgroups Based on Hormone Receptors

**Figure 3:**
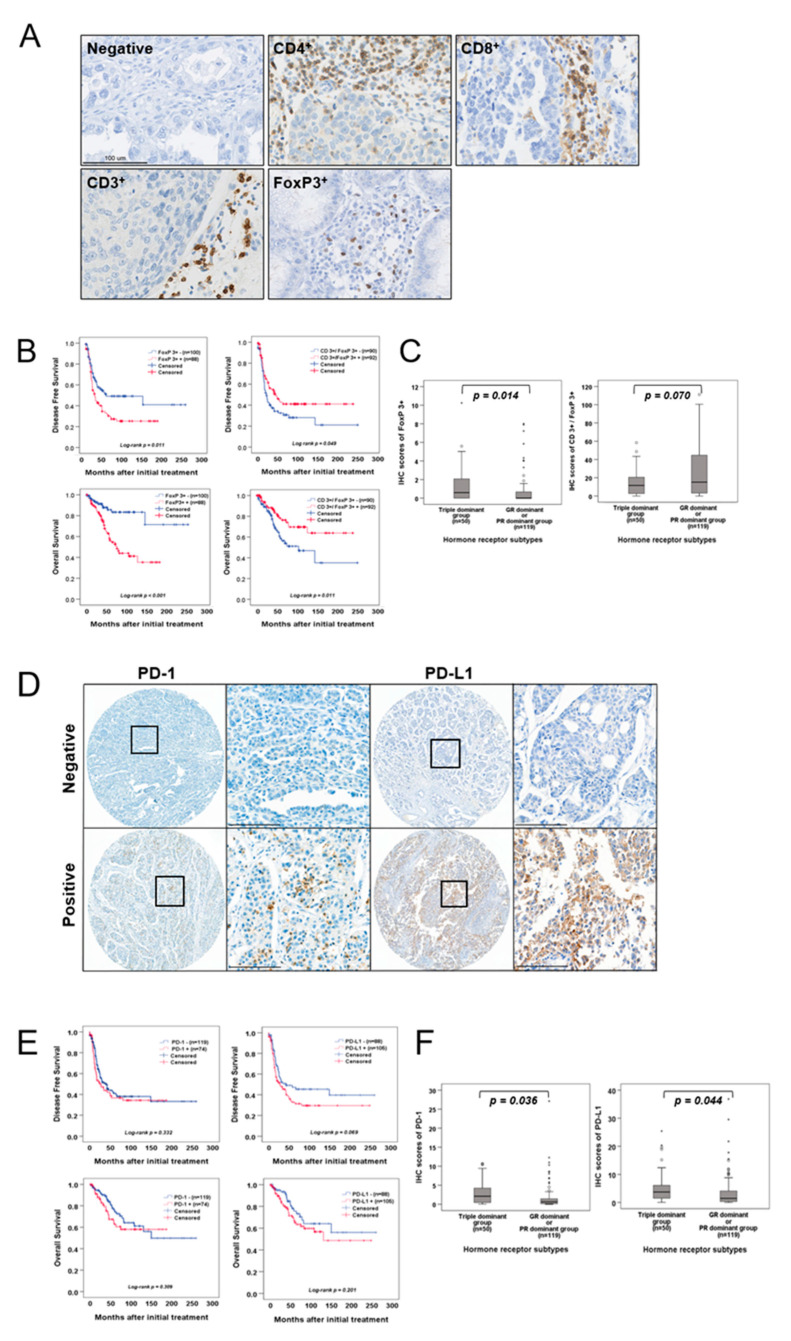
PD-1 and PD-L1 expression in EOC and their association with tumor-infiltrating lymphocytes and EOC subgroups clustered by hormone receptor expression pattern. (**A**) Representative immunohistochemical images of CD4^+^, CD8^+^, CD3^+^, and FoxP3^+^ TILs in EOCs. The TILs were observed in cancer stroma (scale bar: 100 μm). (**B**) DFS and OS of patients with EOC were based on FoxP3^+^ TIL infiltration level and CD3^+^/FoxP3^+^ ratio. (**C**) A boxplot of FoxP3^+^ TIL infiltration level or CD3^+^/FoxP3^+^ ratio and subgroups clustered by hormone receptor expression pattern. (**D**) Representative immunohistochemical images of PD-1 and PD-L1 levels in EOCs (scale bar: 100 μm). (**E**) The DFS and OS of patients with EOC were based on PD-1 and PD-L1 expression. (**F**) A boxplot of TIL infiltration with PD-1 or PD-L1 expression in subgroups clustered by hormone receptor expression pattern in EOC.

Considering the importance of hormone receptors in the prognosis of EOC, we hypothesized that they contribute to the behavior of TILs, as the nuclear factor (NF)-κB pathway is involved in both hormone receptor signaling and TIL biology [20,21,22,23]. Before verifying this hypothesis, we first evaluated the expression of CD4+, CD8+, CD3+, and FoxP3+ in whole tissue sections of the EOC. Representative images of IHC staining are shown in Figure 3A, and the clinicopathological characteristics are presented in Supplementary Table S2. The prognostic significance of specific subtype of infiltrating TILs and their ratio showed that a high infiltration of FoxP3+ TILs was associated with poor DFS and OS (*p* = 0.011, *p* < 0.001, respectively; Figure 3B) and increased CD3+/FoxP3+ ratio was associated with favorable DFS and OS (*p* = 0.049, *p* = 0.011, respectively; Figure 3B). We further analyzed whether hormone receptor expression might influence the behavior of TILs in EOC and found that FoxP3+ TILs were significantly more abundant in the triple dominant group than in GR- and PR-dominant groups (*p* = 0.014; Figure 3C). In addition, the CD3+/FoxP3+ ratio significantly decreased in the triple dominant group (*p* = 0.070, Figure 3C).

### 2.4. Association between PD-1 or Programmed Cell Death Protein-Ligand1 (PD-L1) Expression and EOC Subgroups Based on Hormone Receptors

As recognizing FoxP3+ alone does not sufficiently define Tregs, we also performed a similar analysis to determine the association between PD-1 or PD-L1 expression and the triple dominant group, a subgroup of EOC. Before verifying their association, we first evaluated PD-1 or PD-L1 expression in EOC. A representative IHC image is shown in Figure 3D, and clinicopathological characteristics are presented in Supplementary Table S2. Neither PD-1 nor PD-L1 revealed a significant association with DFS (*p* = 0.322, *p* = 0.069, respectively; Figure 3E) or OS (*p* = 0.309, *p* = 0.201, respectively; Figure 3E). Importantly, when evaluating the association between the triple dominant group and PD-1, and PD-L1 expression, we observed a significantly upregulated PD-1 and PD-L1 expression compared with that of other groups (*p* = 0.036, *p* = 0.044, respectively; Figure 3F).

### 2.5. NanoString Analysis of the Triple Dominant Group

**Figure 4:**
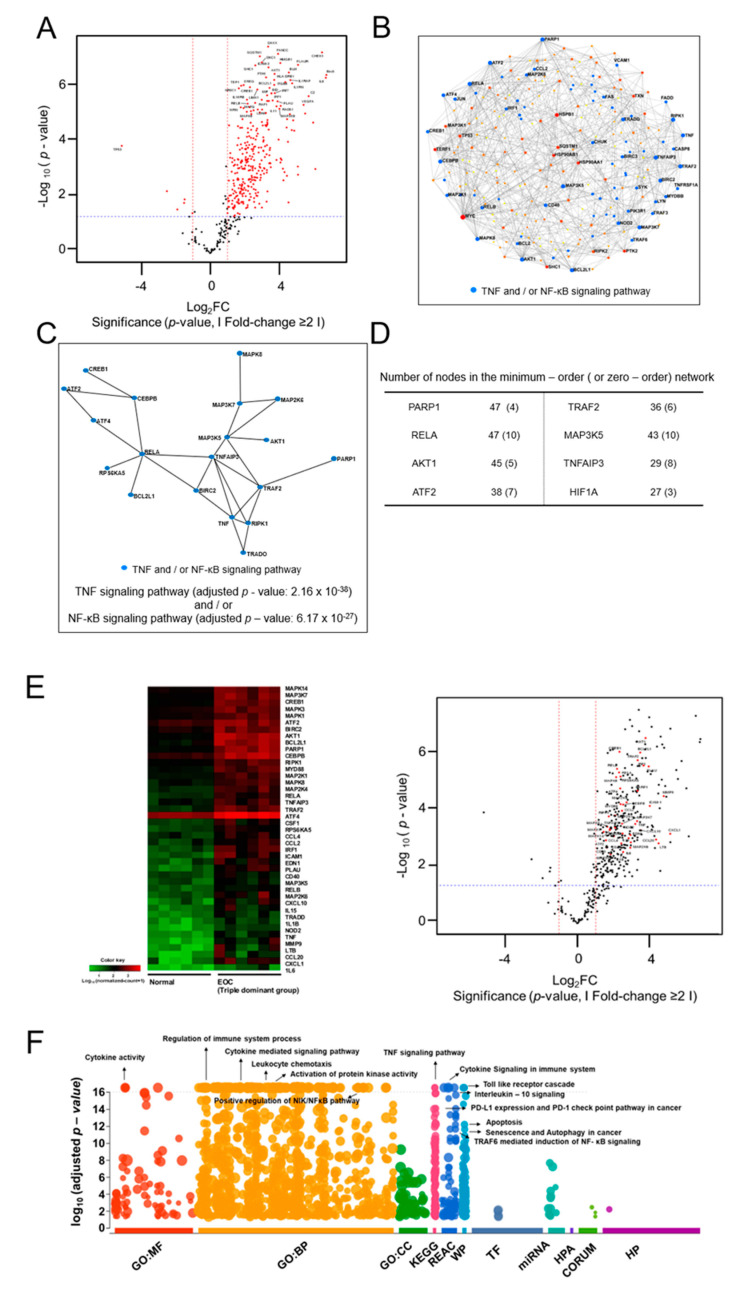
Analysis of differentially expressed genes (DEGs) in the triple dominant EOC group as compared to gene expression in normal ovarian epithelial tissues. Analysis of differentially expressed genes (DEGs) in the triple dominant EOC group compared with gene expression in normal ovarian epithelial tissues. (**A**) Volcano plot shows the upregulated DEGs in the triple dominant EOC group compared with gene expression in normal ovarian epithelial tissues. (**B**) Minimum-order and (**C**) zero-order protein-protein interaction (PPI) network was constructed with 167 upregulated DEGs using NetworkAnalyst (https://www.networkanalyst.ca/, accessed on 15 December 2020) (**D**) Genes with a higher number of PPI interactions are listed. (**E**) Most significantly upregulated pathways identified in the PPI networks are shown using Heatmap and volcano plot. (**F**) g:Profiler (https://biit.cs.ut.ee/gprofiler/, accessed on 15 December 2020) was used to dissect molecular pathways of 167 upregulated DEGs in the triple dominant EOC group compared with gene expression in normal ovarian epithelial tissues with the following categories: [GO:MF—molecular function; GO:BP—biologic process; GO:CC—cellular component; KEGG—Kyoto Encyclopedia of Genes and Genomes; REAC—reactome; WP—Wikipathways; TF—TRANSFAC; miRNA—miRTarBase; HPA—human protein atlas; CORUM—CORUM protein complexes; HP—human phenotype ontology].

We then analyzed the DEGs with mRNA extracted from six normal ovarian epithelial tissues and six EOC tissues in the triple dominant EOC group to gain insight into the molecular details of the triple dominant group [24]. A total of 228 upregulated DEGs were identified with an adjusted *p*-value cut-off of 0.05 (Figure 4A, Supplementary Table S3). Expectedly, several upregulated genes were classified as cytokine-related genes. To gain further biological insights, we constructed ovarian tissue-specific PPI networks using NetworkAnalyst [25] with the identified upregulated DEGs (n = 169). The results showed that γ-rel avian reticuloendotheliosis viral oncogene homolog A (*RELA*), mitogen-activated protein kinase 5 (*MAP3K5)*, tumor necrosis factor α-induced protein 3 (*TNFAIP3*), BCL2-like protein 1 *(BCL2L1*), receptor-interacting protein kinase 1 (*RIPK1*), tumor necrosis factor receptor-associated factor 2 (*TRAF2*), *PARP1*, and v-akt murine thymoma viral oncogene homolog 1 (*AKT1*) were the most highly connected genes in the minimum-order network (Figure 4B). Moreover, *RELA*, *MAP3K5*, and *TNFAIP3* showed the highest connection in the zero-order network (Figure 4C,D). Gene Ontology analysis of the network components also indicated that genes involved in the TNF (adjusted *p*-value < 2.16 × 10^−38^) and/or NF-κB (adjusted *p*-value < 6.17 × 10^−27^) signaling pathways were significantly enriched with respect to the upregulated DEGs (Figure 4E). Overall, the DEG and PPI network analyses indicated that *RELA*, *MAP3K5*, *TNFAIP3*, *BCL2L1*, *RIPK1*, *TRAF2*, *PARP1*, and *AKT1* profoundly influenced the triple dominant group of EOC through the TNF and/or NF-κB signaling pathways. We also conducted a comprehensive Gene Ontology analysis using g:profiler [26] to analyze the enriched pathways in the triple dominant group of EOC. As expected, many genes were found to be involved in cytokine signaling and the immune system (cytokine receptor binding (adjusted *p*-value < 2.44 × 10^−87^), chemokine activity (adjusted *p*-value < 1.31 × 10^−32^), immune receptor activity (adjusted *p*-value < 3.77 × 10^−12^), and chemokine-mediated signaling pathway (adjusted *p*-value < 7.10 × 10^−38^), (Figure 4E and Supplementary Table S4). Interestingly, we found that some pathways were related to the differentiation of Treg such as positive regulation of T cell activation (adjusted *p*-value < 2.36 × 10^−27^), selective expression of chemokine receptors during T cell polarization (adjusted *p*-value < 1.62 × 10^−18^), and interleukin-2 signaling pathway (adjusted *p*-value < 7.69 × 10^−23^) (Figure 4E and Supplementary Table S4). Moreover, enrichment of interleukin-1 receptor binding (adjusted *p*-value < 1.22 × 10^−30^), response to lipopolysaccharide (adjusted *p*-value < 7.21 × 10^−51^), interleukin-4 and interleukin-13 signaling (adjusted *p*-value < 2.10 × 10^−36^), interleukin-10 signaling (adjusted *p*-value < 2.29 × 10^−33^), toll-like receptor cascades (adjusted *p*-value < 3.18 × 10^−25^), and apoptosis (adjusted *p*-value < 6.67 × 10^−14^) which were responsible for modulating Treg functions were upregulated in the triple dominant group (Figure 4E and Supplementary Table S4). Overall, this finding indicates that the triple dominant group activates host immune system and contributes to the expansion and differentiation of Treg FoxP3+ lymphocytes (Supplementary Table S4) [27,28,29].

## 3. Discussion

The endocrine organs and hormone receptor expression may be associated with the occurrence, progression, and overall prognosis of EOC. However, previous studies have primarily focused on investigating the prognostic value of a single hormone receptor. Although Feng et al. [30] and Kruchten et al. [31] performed cluster analysis over a single isoform of hormone receptors, their results were less conclusive. Moreover, studies involving cluster analysis using steroid hormone receptors, such as GR and AR, in addition to ERα, PR, and ERβ in EOC, are lacking. Meanwhile, herein we demonstrated that the triple dominant (AR^+^/PR^+^/GR^+^) subgroup represents an independent prognostic factor in the multivariate analysis, whereas groups with single dominant steroid hormone receptors did not yield significant results. Taken together, our results suggest that the triple dominant subgroup may play an important role in predicting the treatment and prognosis of EOC.

Interestingly, we found an association between PR activity and EOC prognosis. In general, PR is considered a good prognostic factor for EOC. However, its activity was altered based on which hormone receptors were simultaneously expressed. For example, in the triple dominant subgroup where GR and AR were co-expressed, PR overexpression was associated with poor prognosis, unlike in the PR- or GR-dominant subgroups of EOC (Supplementary Figure S3). Similarly, PR activation is associated with the increased occurrence and progression of breast cancer, whereas GR is related to growth suppression and differentiation. Ogara et al. [32] reported that GR negatively regulates PR activity by modulating PR target genes, as GR and PR share structural and functional similarities. They also suggested that GR-PR heterocomplex formation increases negative regulation of PR activity [33]. In addition to PR, GR regulates the expression of AR target genes, while AR and PR have 88% sequence homology [34,35]. Therefore, we hypothesized that GR might modulate both AR and PR directly and indirectly. The role of hormone receptor crosstalk in cancer is becoming increasingly relevant, although the mechanisms involved remain controversial, particularly in EOC. Thus, our findings suggest the possibility of different transcriptional outcomes for the triple dominant group in EOC. Further investigation in this regard could explain the prognosis associated with the triple dominant group in more detail and contribute to the development of new endocrine combined therapies in the future.

To date, several studies on EOC have reported that an elevated number of Tregs significantly correlates with a worse prognosis [36]. Nevertheless, evaluating TILs in the context of hormone receptors is important as altered expression of hormones and their cognate receptors represent mediators of immune trafficking and inflammatory processes affecting the TME. For instance, several important mechanisms modulated by GR related to apoptosis and differentiation and proliferation of Tregs have been reported. One such mechanism involves GR enhancing the expression of immunosuppressive cytokines, IL-10 and TGF-β, which are capable of subsequently augmenting FoxP3^+^ expression [37,38]. Furthermore, Huang et al. [39] reported that simultaneous overexpression of *BCL-2* and GR effectively inhibits glucocorticoid-dependent apoptosis of Tregs. Moreover, Bereshchenko et al. [40] demonstrated that following its translocation to the nucleus, GR induces glucocorticoid-induced leucine zipper (GILZ) production, a protein induced by glucocorticoid, as well as enhanced FoxP3^+^ differentiation in naive T cells. Furthermore, GR- or AR-biding regions have been identified in FoxP3^+^, which may modulate the activities of FoxP3^+^Treg cells [41,42]. However, functional studies to better understand the molecular mechanisms associated with the triple dominant subgroup of EOC are necessary. In addition, we plan to incorporate larger numbers of specific histological subgroups to generalize our findings, as we did not find a significant difference in prognosis in the triple dominant group depending on FoxP3^+^ expression, which is a limitation of our study.

The TNF and/or the NF-κB signaling pathways are key regulators of innate and adaptive immune responses, functioning to regulate cell death and survival [36,43]. RELA is a subunit of the NF-κB signaling pathway, while MAP3K5 functions as a positive regulator by phosphorylating IKKβ. Moreover, *BCL2L1*, *RIPK1*, *TRAF2*, *PARP1*, and *AKT1* are protooncogenes upregulated by the NF-κB signaling pathway. Additionally, *TNFAIP3* is an antiapoptotic gene with a rapidly upregulated expression upon activation of the TNF and/or NF-κB signaling pathway [36,44]. Furthermore, *TNFAIP3* is constitutively expressed by T and B cells to inhibit Treg lymphocytes and hyperactivate Th and cytotoxic T cells [44,45]. However, in EOC, the role of *TNFAIP3* remains controversial. Therefore, future studies should evaluate its role in EOC and incorporate the role of *TNFAIP3* with the triple dominant subgroup of EOC to identify its potential as a prognostic or therapeutic target.

Lastly, as the abundance of Tregs and their functionality have been associated with steroid hormone receptors, reducing the expression of these receptors could subsequently reduce the Treg population, which may improve patient survival. Generail et al. [46,47] performed a phase II randomized controlled trial of letrozole with, or without, the immunomodulatory agent cyclophosphamide in patients with breast cancer. Results showed a significant reduction of Treg number and a significant correlation between the reduced number of Tregs and the number of patients who achieved complete remission when treated with letrozole in combination with cyclophosphamide, as estrogen was shown to increase Treg number and functionality. Therefore, in the triple dominant subgroup, inhibiting AR, PR, and GR via combination immunotherapy may improve the number of patients with EOC achieving complete remission.

In conclusion, we identified an EOC subgroup based on hormone receptor expression that displays prognostic significance. Moreover, our newly constructed EOC subgroup classification revealed important TIL features. Although additional studies are necessary to clarify the underlying mechanism, our newly classified triple dominant EOC subgroup has prognostic and predictive value. Overall, the proposed hormone receptor expression-based classification may contribute to precision medicine development in EOC and determine the most efficient therapeutic course.

## 4. Materials and Methods

### 4.1. Patients and Tumor Specimens

A total of 212 EOC, 57 borderline ovarian tumor, 153 benign epithelial ovarian tumors and 79 nonadjacent normal epithelial tissue samples were obtained from patients who underwent primary surgery at the Gangnam Severance Hospital between 2004 and 2012, and some of the samples were obtained from the Korea Gynecologic Cancer Bank as part of the Bio and Medical Technology Development Program of the Ministry of the National Research Foundation (NRF), funded by the Korean government (MIST) (NRF-2017M3A9B8069610). The International Federation of Gynecology and Obstetrics (FIGO) classification was used for tumor staging, and clinical information, including surgical procedure, survival time, survival status, and age, were collected by reviewing the medical records of the patients. The patients’ response to therapy was assessed by using computed tomography with Response Evaluation Criteria in Solid Tumors (RECIST; version 1.1). Tumor grade and cell type were evaluated by reviewing pathological reports, and all tumor samples were histologically examined by two gynecologic pathologists. All biological samples were collected after obtaining informed consent from participants, following the guidelines of the institutional review board (IRB) of the Gangnam Severance Hospital (IRB No. 3-2018-0122).

### 4.2. Tissue Microarray and Immunohistochemistry

Tissue microarray (TMA) blocks comprising tissue cores (1 mm) with a sufficient proportion of tumor cells obtained from formalin-fixed paraffin-embedded (FFPE) tumor tissue blocks were used for immunohistochemistry (IHC) analysis of ERα, AR, glucocorticoid receptor (GR), PR, ERβ, PD-1, and PD-L1 (5-μm-thick sections cut using a rotary microtome), whereas whole tissue sections were used for the IHC analysis of CD4+, CD8+, CD3+, and FoxP3+. The sections were deparaffinized and rehydrated with graded ethanol. Then, the sections were treated with 3% H_2_O_2_ solution in methanol for 30 min to suppress endogenous peroxidase activity. Thereafter, heat-induced antigen retrieval was performed by incubating the sections in a target retrieval buffer at pH 6.0 (Dako, Carpinteria, CA, USA) using a steam pressure cooker (Pascal; Dako) for 20 min, and the slides were stained with the primary antibodies listed in Supplementary Table S5 with Autostainer Plus (Dako) for 1 h at room temperature. Then, EnVision+ Dual Link System-HRP (Dako) and DAB+ (3,3′-diaminobenzidine; Dako) were used for the visualization of antigen-antibody reactions. After dehydrating and counterstaining with hematoxylin, the slides were mounted in Faramount Aqueous Mounting Medium (Dako). Proper positive and negative controls were included.

### 4.3. Evaluation of Immunohistochemical (IHC) Staining

The stained TMA sections were scanned with a high-resolution optical scanner (NanoZoomer 2.0 HT; Hamamatsu Photonics K.K., Hamamatsu City, Japan) at 20× objective magnification (0.5 μm resolution). In the case of ERα, AR, GR, PR, and ERβ, the scanned sections were analyzed using Visiopharm software, version 4.5.1.324 (VIS; Visiopahrm, Hφrsholm, Denmark). Two hundred and seven EOC, 56 borderline ovarian tumor, 110 benign tumor, and 79 nonadjacent normal epithelial tissues for ERα expression; 192 EOC, 55 borderline ovarian tumor, 111 benign tumor, and 79 nonadjacent normal epithelial tissues for AR expression; 208 EOC, 54 borderline ovarian tumor, 112 benign tumor, and 78 nonadjacent normal epithelial tissues for PR expression; 209 EOC, 57 borderline ovarian tumor, 123 benign tumor, and 79 nonadjacent normal epithelial tissues for GR expression; and 205 EOC, 55 borderline ovarian tumor, 108 benign tumor, and 79 nonadjacent normal epithelial tissues for ERβ expression were analyzed using the TMA. The staining intensity was scored semi-quantitatively using the immunoreactive score (IRS) with a predefined algorithm and settings. The IRS was obtained as the product of immunostaining intensity (0 = negative, 1 = weak, 2 = moderate, 3 = strong), while the overall immunostaining score was calculated by multiplying the percentage of positive cells and immunostaining intensity (possible range: 0–300). For PD-1 and PD-L1 scoring, tumor areas were automatically outlined, followed by manual editing to exclude necrotic tissues, and PD-1 and PD-L1 levels were quantified by analyzing the percentage of DAB+ using Visiopharm software version 4.5.1.324 (VIS; Visiopharm). Lastly, TILs were evaluated following the recommendation of the International TILs Working Group [48]. Six independent regions of interest (ROI with the highest TIL numbers, comprising 2 mm^2^ stromal areas with the highest number of TILs from entire tumor sections, were selected and evaluated for TILs using Visiopharm software version 4.5.1.324 (VIS; Visiopharm). The area for stromal TIL evaluation was set within the invasive tumor borders that did not include immune infiltration in the adjacent normal tissue of EOCs. Additionally, mononuclear stromal cells which were not directly contacting with cancer cells were considered stromal TILs. Positive staining was assessed quantitatively, and the results expressed as a percentage of positively stained area relative to the total ROI. The results for each section’s ROIs were then averaged.

### 4.4. mRNA-seq Data Analysis

The gene expression profiling data consisting of 185 primary ovarian tumors and 10 normal ovarian surface epithelia profiled by using the Affymetrix human U133A microarray were downloaded from the gene expression omnibus (GEO) data (http://www.ncbi.nlm.nih.gov/geo/query/acc.cgi?acc=GSE26712, GSE26712, accessed on 30 April 2021).

### 4.5. RNA Extraction and Quality Control

FFPE slides of normal ovarian epithelial tissues and EOCs were stained with hematoxylin and eosin to identify and mark normal ovarian epithelial tissues and EOC regions by a gynecological pathologist. In the case of EOC, FFPE sections consisting of more than 85% tumor cells within each specimen were subjected to laser-capture microdissection (LCM). After sectioning FFPE tissues, they were placed on slides coated with polyethylene terephthalate membrane (Leica Microsystems Inc., IL, USA), and LCM was performed using the Leica AS LMD laser microdissection system (Leica Microsystems Inc.) following the manufacturer’s instructions. Total RNA was extracted using the RNeasy FFPE kit (Qiagen, Valencia, CA), according to the manufacturer’s instructions. RNA concentration was quantified using a Nanodrop spectrophotometer (Thermo Scientific, Waltham, MA, USA), and its quality was verified on formaldehyde agarose gels.

### 4.6. Quantification of Gene Expression and Analysis of Differentially Expressed Genes (DEGs) Using the NanoString nCounter Platform

Three hundred nanograms of isolated RNA from each sample were used for hybridization with the PanCancer IO 360 Panel Gene Expression Panel (NanoString Technologies, Inc., Seattle, WA, USA), according to the manufacturer’s instructions and transferred to the Digital Analyzer (NanoString Technologies, Inc.) for analysis. After background correction, gene expression was normalized using the target to housekeeping gene expression ratio, with nSolver Analysis Software version 4.0 (NanoString Technologies, Inc.). Finally, DEGs between the normal ovarian epithelial tissues and EOCs were log_2_ transformed. Group analyses for DEGs were performed using in-house R scripts. The scatter plots for the gene expression values, volcano plots for the expression fold-changes, and *p*-values between the two selected samples were similarly calculated using in-house R scripts.

### 4.7. Network Analysis

To construct the ovarian tissue-specific protein-protein interaction (PPI) network with the minimum- or zero-order network model, 169 upregulated DEGs were used as an input for network analyst (https://www.networkanalyst.ca/, accessed on 15 December 2020) [25].

### 4.8. Gene Ontology Analysis

All the genes listed in the Nanostring analysis were used to perform g:profiler (https://biit.cs.ut.ee/gprofiler/gost, accessed on 30 April 2021) with default parameters to investigate the affected pathways [26]. The analyzed parameters for the statistical domain scope included “g:SCS threshold” for the significance threshold and 0.05 as a user threshold, and the statistical domain scope was set as custom.

### 4.9. Statistical Analysis

Statistical analyses of hormone receptors, TILs, PD-1, and PD-L1 expression data were performed using either Mann–Whitney U-test or Kruskal–Wallis test, as appropriate. Kaplan–Meier method was used to assess disease-free survival (DFS) and overall survival (OS). Survival was analyzed using the log-rank test with the cut-off values that had the highest discriminative power. A clustering analysis was performed to identify EOC samples with similar hormone receptor expression patterns. Cox proportional hazards model was used to estimate the hazard ratios (HR) and confidence intervals (CIs) in both univariate and multivariate models. Statistical analyses were performed using either SPSS version 25.0 (SPSS, Chicago, IL) or R software (version 3.6.3). Results with a *p*-value < 0.05 were considered statistically significant.

## Figures and Tables

**Table 1:** Univariate and multivariate analyses of the association between prognostic variables and disease-free survival rates in epithelial ovarian cancer.

Variables	Univariate Analysis		Multivariate Analysis	
	Hazard Ratio [95%CI *]	*p*-Value	Hazard Ratio [95%CI *]	*p*-Value
FIGO stage (III-IV)	6.427 [3.335–12.386]	<0.001	4.604 [2.260–9.380]	<0.001
Cell type (Serous)	0.337 [0.202–0.561]	<0.001	0.525 [0.294–0.939]	0.030
Tumor grade (Poor)	1.949 [1.286–2.954]	0.002	1.646 [1.074–2.518]	0.022
CA 125 + (>35 U/mL)	2.397 [1.208–4.753]	0.012	1.004 [0.471–2.141]	0.992
Age (>50)	1.577 [1.064–2.339]	0.023	1.231 [0.810–1.872]	0.330
ERα + ^a^	1.625 [1.038–2.542]	0.034	1.363 [0.842–2.207]	0.207
AR + ^b^	1.887 [1.245–2.860]	0.002	1.546 [0.998–2.396]	0.051
PR + ^c^	1.201 [0.794–1.815]	0.386	NA	
GR + ^d^	2.610 [1.143–5.958]	0.023	1.892 [0.823–4.351]	0.133
ERβ + ^e^	1.824 [1.226–2.714]	0.003	1.403 [0.918–2.543]	0.118
Heatmap (Triple dominant)	2.668 [1.728–4.118]	<0.001	2.176 [1.361–3.487]	0.001

^a^ cut-off value of ERα is over 49.2 of IHC score; ^b^ cut-off value of AR is over 10.85 of IHC score; ^c^ cut-off value of PR is over 21.18 of IHC score; ^d^ cut-off value of GR is over 8.65 of IHC score; ^e^ cut-off value of ERβ is over 105.97 of IHC score; * CI, confidence interval; FIGO, International Federation of Gynecology and Obstetrics; NA: Not available.

## Data Availability

The data that support the findings of this study are available from the corresponding author, upon reasonable request.

## Supplementary Materials

The following are available online at https://www.mdpi.com/article/10.3390/ijms22115714/s1.

## References

[1] Torre L.A., Trabert B., DeSantis C.E., Mph K.D.M., Samimi G., Runowicz C.D., Gaudet M.M., Jemal A., Siegel R.L. (2018). Ovarian cancer statistics. CA A Cancer J. Clin..

[2] Hennessy B.T., Coleman R.L., Markman M. (2009). Ovarian cancer. Lancet.

[3] Weiss L., Huemer F., Mlineritsch B., Greil R. (2016). Immune checkpoint blockade in ovarian cancer. Memo-Mag. Eur. Med Oncol..

[4] Zhang L., Conejo-Garcia J.R., Katsaros D., Gimotty P.A., Massobrio M., Regnani G., Makrigiannakis A., Gray H., Regnani G., Schlienger K. (2003). Intratumoral T cells, recurrence, and survival in epithelial ovarian cancer. N. Engl. J. Med..

[5] Gasparri M.L., Attar R., Palaia I., Perniola G., Marchetti C., Di Donato V., Farooqi A.A., Papadia A., Panici P.B. (2015). Tumor infiltrating lymphocytes in ovarian cancer. Asian Pac. J. Cancer Prev..

[6] Wouters M.C.A., Komdeur F.L., Workel H.H., Klip H.G., Plat A., Kooi N.M., Wisman G.B.A., Mourits M.J.E., Arts H.J.G., Oonk M.H.M. (2016). Treatment Regimen, Surgical Outcome, and T-cell Dif-ferentiation Influence Prognostic Benefit of Tumor-Infiltrating Lymphocytes in High-Grade Serous Ovarian Cancer. Clin. Cancer Res..

[7] Ou Y., Cannon M.J., Nakagawa M. (2018). Regulatory T Cells in Gynecologic Cancer. MOJ Immunol..

[8] Mizushima T., Miyamoto H. (2019). The Role of Androgen Receptor Signaling in Ovarian Cancer. Cells.

[9] McDonnel A.C., Van Kirk E.A., Isaak D.D., Murdoch W.J. (2003). Inhibitory Effects of Progesterone on Plasma Membrane Fluidity and Tumorigenic Potential of Ovarian Epithelial Cancer Cells. Exp. Biol. Med..

[10] Ho S.-M. (2003). Estrogen, Progesterone and Epithelial Ovarian Cancer. Reprod. Biol. Endocrinol..

[11] Coenen C.M., Thomas C.M., Borm G.F., Hollanders J.M., Rolland R. (1996). Changes in androgens during treatment with four low-dose contraceptives. Contraception.

[12] Arias-Pulido H., Smith H.O., Joste N.E., Bocklage T., Qualls C.R., Chavez A., Prossnitz E.R., Verschraegen C.F. (2009). Estrogen and progesterone receptor status and outcome in epithelial ovarian cancers and low malignant potential tumors. Gynecol. Oncol..

[13] Pierdominici M., Maselli A., Colasanti T., Giammarioli A.M., Delunardo F., Vacirca D., Sanchez M., Giovannetti A., Malorni W., Ortona E. (2010). Estrogen receptor profiles in human peripheral blood lymphocytes. Immunol. Lett..

[14] Kovats S. (2015). Estrogen receptors regulate innate immune cells and signaling pathways. Cell. Immunol..

[15] Mantalaris A., Panoskaltsis N., Sakai Y., Bourne P., Chang C., Messing E.M., Wu J.H.D. (2001). Localiza-tion of androgen receptor expression in human bone marrow. J. Pathol..

[16] Arruvito L., Giulianelli S., Flores A.C., Paladino N., Barboza M., Lanari C., Fainboim L. (2008). NK cells expressing a progesterone receptor are susceptible to progesterone-induced apoptosis. J. Immunol..

[17] Dosiou C.E., Hamilton A., Pang Y., Overgaard M.T., Tulac S., Dong J., Thomas P., Giudice L.C. (2007). Expression of membrane progesterone receptors on human T lymphocytes and Jurkat cells and activation of G-proteins by progesterone. J. Endocrinol..

[18] Ghebeh H., Mohammed S., Al-Omair A., Qattant A., Lehe C., Al-Qudaihi G., Elkum N., Al-shabanah M., Bin Amer S., Tulbah A. (2006). The B7-H1 (PD-L1) T Lymphocyte-Inhibitory Molecule Is Expressed in Breast Cancer Patients with Infiltrating Ductal Carcinoma: Correlation with Important High-Risk Prognostic Factors. Neoplasia.

[19] Duma D., Jewell C.M., Cidlowski J.A. (2006). Multiple glucocorticoid receptor isoforms and mechanisms of post-translational modification. J. Steroid Biochem. Mol. Biol..

[20] Ling J., Kumar R. (2012). Crosstalk between NFkB and glucocorticoid signaling: A potential target of breast cancer therapy. Cancer Lett..

[21] Anderson D.M., Maraskovsky E., Billingsley W.L., Dougall W.C., Tometsko M.E., Roux E.R., Teepe M.C., DuBose R.F., Cosman D., Galibert L.J. (1997). A homologue of the TNF receptor and its ligand en-hance T-cell growth and dendritic-cell function. Nat. Cell Biol..

[22] Frasor J., Weaver A., Pradhan M., Dai Y., Miller L.D., Lin C.Y., Stanculescu A. (2009). Positive cross-talk between estro-gen receptor and NF-kappaB in breast cancer. Cancer Res..

[23] Burg V.D., der Saag P.T.V. (1996). Endocrinology and paracrinology: Nuclear factor-kappa-B/steroid hormone receptor interactions as a functional basis of anti-inflammatory action of steroids in reproductive organs. Mol. Hum. Reprod..

[24] Love M.I., Huber W., Anders S. (2014). Moderated estimation of fold change and dispersion for RNA-seq data with DESeq2. Genome Biol..

[25] Zhou G., Soufan O., Ewald J., Hancock R.E.W., Basu N., Xia J. (2019). NetworkAnalyst 3.0: A visual ana-lytics platform for comprehensive gene expression profiling and meta-analysis. Nucleic Acids Res..

[26] Raudvere U., Kolberg L., Kuzmin I., Arak T., Adler P., Peterson H., Vilo J. (2019). g:Profiler: A web server for functional enrichment analysis and conversions of gene lists (2019 update). Nucleic Acids Res..

[27] Sutmuller R.P., Morgan M.E., Netea M.G., Grauer O., Adema G.J. (2006). Toll-like receptors on regulatory T cells: Expanding immune regulation. Trends Immunol..

[28] Hoeppli R.E., Wu D., Cook L., Levings M.K. (2015). The Environment of Regulatory T Cell Biology: Cyto-kines, Metabolites, and the Microbiome. Front. Immunol..

[29] Kitagawa Y., Sakaguchi S. (2017). Molecular control of regulatory T cell development and function. Curr. Opin. Immunol..

[30] Feng Z., Wentao Y., Bi R., Xiaojun C., Chen X., Yang W., Wu X. (2016). A clinically applicable molecular classification for high-grade serous ovarian cancer based on hormone receptor expression. Sci. Rep..

[31] Van Kruchten M., van der Marel P., de Munck L., Hollema H., Arts H., Timmer-Bosscha H., de Vries E., Hospers G., Reyners A. (2015). Hormone receptors as a marker of poor survival in epithelial ovarian cancer. Gynecol. Oncol..

[32] Ogara M.F., A Rodríguez-Seguí S., Marini M., Nacht A.S., Stortz M., Levi V., Presman D., Vicent G.P., Pecci A. (2019). The glucocorticoid receptor interferes with progesterone receptor-dependent genomic regula-tion in breast cancer cells. Nucleic Acids Res..

[33] Wan Y., Nordeen S.K. (2003). Overlapping but distinct profiles of gene expression elicited by glucocorti-coids and progestins. Recent Prog. Horm. Res..

[34] Beato M., Chávez S., Truss M. (1996). Transcriptional regulation by steroid hormones. Steroids.

[35] De Stefano I., Zannoni G.F., Prisco M.G., Fagotti A., Tortorella L., Vizzielli G., Mencaglia L., Scambia G., Gallo D. (2011). Cytoplasmic ex-pression of estrogen receptor beta (ERbeta) predicts poor clinical outcome in advanced serous ovarian cancer. Gynecol. Oncol..

[36] Curiel T.J., Coukos G., Zou L., Alvarez X., Cheng P., Mottram P., Evdemon-Hogan M., Cone-jo-Garcia J., Zhang L., Burow M. (2004). Specific recruitment of regulatory T cells in ovarian carcinoma fosters immune privilege and predicts reduced survival. Nat. Med..

[37] Stary G., Klein I., Bauer W., Koszik F., Reininger B., Kohlhofer S., Gruber K., Skvara H., Jung T., Stingl G. (2010). Glucocorticosteroids Modify Langerhans Cells To Produce TGF-β and Expand Regulatory T Cells. J. Immunol..

[38] Hou Y., Feng Q., Xu M., Li G.-S., Liu X.-N., Sheng Z., Zhou H., Ma J., Wei Y., Sun Y.-X. (2016). High-dose dexamethasone corrects impaired myeloid-derived suppressor cell function via Ets1 in immune thrombocytopenia. Blood.

[39] Huang S.J., Cidlowski J.A. (2002). Phosphorylation status modulates Bcl-2 function during glucocorticoid-induced apoptosis in T lymphocytes. FASEB J..

[40] Bereshchenko O., Coppo M., Bruscoli S., Biagioli M., Cimino M., Frammartino T., Sorcini D., Ve-nanzi A., Di Sante M., Riccardi C. (2014). GILZ Promotes Production of Peripherally Induced Treg Cells and Mediates the Crosstalk between Glucocorticoids and TGF-β Signaling. Cell Rep..

[41] Ugor E., Prenek L., Pap R., Berta G., Ernszt D., Najbauer J., Németh P., Boldizsár F., Berki T. (2018). Glucocorticoid hormone treatment enhances the cytokine production of regulatory T cells by upregulation of Foxp3 expression. Immunobiology.

[42] Walecki M., Eisel F., Klug J., Baal N., Paradowska-Dogan A., Wahle E., Hackstein H., Meinhardt A., Fijak M. (2015). Androgen receptor modulates Foxp3 expression in CD4+CD25+Foxp3+ regulatory T-cells. Mol. Biol. Cell.

[43] Zhang Y., Tong L., Chen S., Wu W., Wang L. (2018). Analysis of NFKB2mediated regulation of mecha-nisms underlying the development of Hodgkin’s lymphoma. Mol. Med. Rep..

[44] Lee E.G., Boone D.L., Chai S., Libby S.L., Chien M., Lodolce J.P., Ma A. (2000). Failure to regulate TNF-induced NF-kappaB and cell death responses in A20-deficient mice. Science.

[45] Song X.-T., Kabler K.E., Shen L., Rollins L., Huang X.F., Chen S.-Y. (2008). A20 is an antigen presentation attenuator, and its inhibition overcomes regulatory T cell–mediated suppression. Nat. Med..

[46] Generali D., Bates G., Berruti A., Brizzi M.P., Campo L., Bonardi S., Bersiga A., Allevi G., Milani M., Aguggini S. (2009). Immunomodulation of FOXP3+ Regulatory T Cells by the Aromatase Inhibitor Let-rozole in Breast Cancer Patients. Clin. Cancer Res..

[47] Bates G.J., Fox S.B., Han C., Leek R.D., Garcia J.F., Harris A.L., Banham A.H. (2006). Quantification of Regulatory T Cells Enables the Identification of High-Risk Breast Cancer Patients and Those at Risk of Late Relapse. J. Clin. Oncol..

[48] Salgado R., Denkert C., Demaria S., Sirtaine N., Klauschen F., Pruneri G., Wienert S., Eynden G.V.D., Baehner F.L., Penault-Llorca F. (2015). The evaluation of tumor-infiltrating lymphocytes (TILs) in breast cancer: Recommendations by an International TILs Working Group. Ann. Oncol..

