# Supplementary material for: Clinical Significance of Tumor Infiltrating Lymphocytes in Association with Hormone Receptor Expression Patterns in Epithelial Ovarian Cancer
Source: Int J Mol Sci. 2021 May 27;22(11):5714. doi: 10.3390/ijms22115714 (PMC8198528; doi:10.3390/ijms22115714)
Supplement: Supplementary file 1 [file ijms-22-05714-s001.zip › 1. Revision Supplementary Table S1. HR.pdf]

Supplementary Table S1. Clinico pathological characteristics of hormone receptors in epithelial ovarian cancer.

	ERα[a]			AR[b]			PR[c]			GR[d]			ERβ[e]		
	Low n (%)	High n (%)	Total n (%)	Low n (%)	High n (%)	Total n (%)	Low n (%)	High n (%)	Total n (%)	Low n (%)	High n (%)	Total n (%)	Low n (%)	High n (%)	Total n (%)
**Diagnosis**	*p < 0.001*			*p < 0.001*			*p < 0.001*			*p < 0.001*			*p = 0.060*		
**Normal**	75(94.9)	4 (5.1)	79(100)	56(70.9)	23 (29.1)	79(100)	36(46.2)	42(53.8)	78(100)	39(49.4)	40(50.6)	79(100)	33(41.8)	46(58.2)	79(100)
**Benign**	104(94.5)	6(5.5)	110(100)	83(74.8)	28(25.2)	111(100)	53(47.3)	59(52.7)	112(100)	14(11.4)	109(88.6)	123(100)	60(55.6)	48(44.4)	108(100)
**Borderline**	47(83.9)	9(16.1)	56(100)	32(58.2)	23(41.8)	55(100)	37(68.5)	17(31.5)	54(100)	1(1.8)	56(98.2)	57(100)	19(34.5)	36(65.5)	55(100)
**Cancer**	166(80.2)	41(19.8)	207(100)	76(39.6)	116(60.4)	192(100)	153(73.6)	55(26.4)	208(100)	21(10.0)	188(90.0)	209(100)	96(46.8)	109(53.2)	205(100)
**FIGO stage**	*p = 0.134*			*p = 0.092*			*p = 0.953*			*p = 0.050*			*p = 0.047*		
**I-II**	52(86.7)	8(13.3)	60(100)	19(33.9)	37(66.1)	58(100)	43(71.7)	17(28.3)	60(100)	11(18.3)	49(81.7)	60(100)	21(35.6)	38(64.4)	59(100)
**III-IV**	99(77.3)	29(22.7)	128(100)	54(45.8)	64(54.2)	118(100)	91(71.1)	37(28.9)	128(100)	11(8.5)	118(91.5)	129(100)	72(56.7)	55(43.3)	127(100)
**Histology**	*p = 0.027*			*p = 0.126*			*p = 0.008*			*p = 0.391*			*p = 0.682*		
**Serous**	165(81.3)	38(18.7)	203(100)	111(56.9)	84(43.1)	195(100)	120(59.1)	83(40.9)	203(100)	13(6.5)	187(93.5)	200(100)	93(46.7)	106(53.3)	199(100)
**Others**	153(89.5)	18(10.5)	171(100)	81(49.4)	83(50.6)	164(100)	123(72.4)	47(27.6)	170(100)	15(8.9)	154(91.1)	169(100)	81(47.9)	88(52.1)	169(100)
**Tumor grade**	*p = 0.580*			*p = 0.080*			*p = 0.602*			*p = 0.090*			*p = 0.382*		
**Well/Moderate**	73(82.0)	16(18.0)	89(100)	29(34.1)	56(65.9)	85(100)	64(71.9)	25(28.1)	89(100)	15(16.9)	74(83.1)	89(100)	38(42.7)	51(57.3)	89(100)
**Poor**	82(78.8)	22(21.2)	104(100)	41(43.6)	53(56.4)	94(100)	96(75.2)	25(24.8)	101(100)	9(8.7)	94(91.3)	103(100)	50(49.0)	52(51.0)	102(100)
**Histology**	*p = 0.542*			*p = 0.522*			*p = 0.689*			*p =0.140*			*p = 0.689*		
**Low grade Serous**	47(82.5)	10(17.5)	57(100)	38(54.3)	32(45.7)	70(100)	40(70.2)	17(29.8)	57(100)	7(12.3)	50(87.7)	57(100)	28(49.1)	29(50.9)	57(100)
**High grade serous**	61(78.2)	17(21.8)	78(100)	33(60.0)	22(40.0)	55(100)	55(73.3)	20(26.7)	75(100)	4(6.2)	73(94.8)	77(100)	40(52.6)	36(47.4)	76(100)
**CA125**	*p = 0.203*			*p = 0.024*			*p = 0.339*			*p = 0.787*			*p = 0.666*		
**Negative**	102(88.7)	13(11.3)	115(100)	65(60.2)	43(39.8)	108(100)	74(64.9)	40(35.1)	114(100)	9(8.2)	101(91.8)	110(100)	53(47.3)	59(52.7)	112(100)
**Positive (>35U/ml)**	165(83.3)	33(16.7)	198(100)	87(46.5)	100(53.5)	187(100)	137(69.5)	60(30.5)	197(100)	18(9.1)	180(90.9)	198(100)	88(45.6)	105(54.4)	193(100)
**Chemosensitivity**	*p = 0.784*			*p = 0.850*			*p = 0.111*			*p = 0.401*			*p = 0.115*		
**Sensitive**	137(79.2)	36(20.8)	173(100)	64(40.3)	95(59.7)	170(100)	128(71.3)	49(28.7)	171(100)	21(12.2)	151(87.8)	172(100)	76(44.7)	94(52.3)	170(100)
**Resistant**	13(76.5)	4(23.5)	17(100)	7(43.8)	9(56.2)	16(100)	16(88.9)	2(11.1)	18(100)	1(5.6)	17(94.4)	18(100)	11(64.7)	6(35.3)	17(100)

[a]cut-off value of ERα is over 49.2 of IHC score; [b]cut-off value of AR is over 10.85 of IHC score; [c]cut-off value of PR is over 21.18 of IHC score; [d]cut-off value of GR is over 8.65 of IHC score; [e]cut-off value of ERβ is over 105.97 of IHC score; FIGO, International Federation of Gynecology and Obstetrics
