# Supplementary material for: Clinical Significance of Tumor Infiltrating Lymphocytes in Association with Hormone Receptor Expression Patterns in Epithelial Ovarian Cancer
Source: Int J Mol Sci. 2021 May 27;22(11):5714. doi: 10.3390/ijms22115714 (PMC8198528; doi:10.3390/ijms22115714)
Supplement: Supplementary file 1 [file ijms-22-05714-s001.zip › 2. Revision Supplementary Table S2 TIL PD-1 PD -L1.pdf]

Supplementary Table S2. Clinico pathological characteristics of tumor infiltrating lymphocytes. PD-1 and PD-L1 in epithelial ovarian cancer.

Characteristic	CD 4+[a]			CD 8+[b]			CD 4+/CD 8+[c]			CD 3+[d]			FoxP 3+[e]			CD 3+/FoxP 3+[f]			PD-1+ [g]			PD-L1+[h]		
	Low	High	Total	Low	High	Total	Low	High	Total	Low	High	Total	Low	High	Total	Low	High	Total	Low	High	Total	Low	High	Total
	- (n)	+ (n)	n (%)	- (n)	+ (n)	n (%)	- (n)	+ (n)	n (%)	- (n)	+ (n)	n (%)	- (n)	+ (n)	n (%)	- (n)	+ (n)	n (%)	- (n)	+ (n)	n (%)	- (n)	+ (n)	n (%)
**FIGO stage**	$p = 0.021$			$p = 0.051$			$p = 0.071$			$p = 0.033$			$p < 0.001$			$p = 0.621$			$p = 0.016$			$p = 0.018$		
**I-II**	19 (32.2)	40 (67.8)	59 (100)	33 (55.9)	26 (44.1)	59 (100)	52 (96.3)	2 (3.7)	54 (100)	50 (86.2)	8 (13.8)	58 (100)	45 (75.0)	15 (25.0)	60 (100)	26 (46.4)	30 (53.6)	56 (100)	45 (75.0)	15 (25.0)	60 (100)	36 (60.0)	24 (40.0)	60 (100)
**III-IV**	22 (17.2)	106 (82.8)	128 (100)	52 (40.6)	76 (59.4)	128 (100)	105 (87.5)	15 (12.5)	120 (100)	92 (71.9)	36 (28.1)	128 (100)	54 (42.5)	73 (57.5)	127 (100)	63 (46.4)	62 (49.6)	125 (100)	74 (56.1)	58 (43.9)	132 (100)	55 (41.7)	77 (58.3)	132 (100)
**Cell type**	$p = 0.352$			$p = 0.017$			$p = 0.431$			$p = 0.049$			$p < 0.001$			$p = 0.794$			$p = 0.068$			$p = 0.211$		
**Serous**	25 (19.8)	101 (80.2)	126 (100)	50 (39.7)	76 (60.3)	126 (100)	106 (89.1)	13 (10.9)	119 (100)	91 (72.2)	35 (27.8)	126 (100)	53 (42.4)	72 (57.6)	125 (100)	60 (48.8)	63 (51.2)	123 (100)	80 (55.9)	63 (44.1)	143 (100)	63 (43.8)	81 (56.3)	144 (100)
**Others**	16 (25.8)	46 (74.2)	62 (100)	36 (58.1)	26 (41.9)	62 (100)	52 (92.9)	4 (7.1)	56 (100)	52 (85.2)	9 (14.8)	61 (100)	47 (53.2)	16 (25.4)	63 (100)	30 (50.8)	29 (49.2)	59 (100)	47 (69.1)	21 (30.9)	68 (100)	36 (52.9)	32 (47.1)	68 (100)
**Tumor grade**	$p = 0.210$			$p = 0.099$			$p = 0.811$			$p = 0.017$			$p = 0.119$			$p = 0.244$			$p = 0.018$			$p = 0.040$		
**Well/Moderate**	21 (25.9)	60 (74.1)	81 (100)	43 (53.1)	38 (46.9)	81 (100)	67 (89.3)	8 (10.7)	75 (100)	68 (84.0)	13 (16.0)	81 (100)	48 (58.5)	34 (41.5)	82 (100)	35 (45.5)	42 (54.5)	77 (100)	60 (68.2)	28 (31.8)	88 (100)	47 (52.8)	42 (47.2)	89 (100)
**Poor**	16 (18.0)	73 (82.0)	89 (100)	36 (40.4)	53 (59.6)	89 (100)	76 (90.5)	8 (9.5)	84 (100)	60 (68.2)	28 (31.8)	88 (100)	41 (46.6)	47 (53.4)	88 (100)	48 (54.5)	40 (45.5)	88 (100)	54 (51.4)	51 (48.6)	105 (100)	40 (38.1)	65 (61.9)	105 (100)
**CA125**	$p < 0.001$			$p = 0.012$			$p = 0.286$			$p = 0.477$			$p = 0.285$			$p = 0.042$			$p = 0.561$			$p = 0.290$		
**Negative**	15 (53.6)	13 (46.4)	28 (100)	19 (67.9)	9 (32.1)	28 (100)	24 (96.0)	1 (4.0)	25 (100)	22 (81.5)	5 (18.5)	27 (100)	18 (62.1)	11 (37.9)	29 (100)	18 (66.7)	9 (33.3)	27 (100)	23 (65.7)	12 (34.3)	35 (100)	19 (54.3)	16 (45.7)	35 (100)
**Positive (>35 U/mL)**	26 (16.6)	131 (83.4)	157 (100)	66 (42.0)	91 (58.0)	157 (100)	131 (89.1)	16 (10.9)	147 (100)	118 (75.2)	39 (24.8)	157 (100)	80 (51.3)	76 (48.7)	156 (100)	69 (45.4)	83 (54.6)	152 (100)	104 (60.5)	68 (39.5)	172 (100)	77 (44.5)	96 (55.5)	173 (100)
**Chemosensitivity**	$p = 0.216$			$p = 0.287$			$p = 0.635$			$p = 0.758$			$p = 0.375$			$p = 0.576$			$p = 0.355$			$p = 0.594$		
**Sensitive**	33 (20.9)	125 (79.1)	128 (100)	67 (42.4)	91 (57.6)	158 (100)	129 (89.0)	16 (11.0)	145 (100)	118 (75.2)	39 (24.8)	157 (100)	77 (48.7)	81 (51.3)	158 (100)	75 (49.3)	77 (50.7)	152 (100)	104 (59.1)	72 (40.9)	176 (100)	80 (45.5)	96 (54.5)	176 (100)
**Resistant**	1 (7.1)	13 (92.9)	14 (100)	8 (57.1)	6 (42.9)	14 (100)	13 (92.9)	1 (7.1)	14 (100)	10 (71.4)	4 (28.6)	14 (100)	8 (61.5)	5 (38.5)	13 (100)	8 (57.1)	6 (42.9)	14 (100)	12 (70.6)	5 (29.4)	17 (100)	7 (38.9)	11 (61.1)	18 (100)
**Hormone receptor**	$p = 0.656$			$p = 0.258$			$p = 0.389$			$p = 0.831$			$p = 0.008$			$p < 0.001$			$p = 0.001$			$p = 0.002$		
**Triple dominant**	11 (26.8)	30 (73.2)	41 (100)	16 (39.0)	25 (61.0)	41 (100)	37 (94.9)	2 (5.1)	39 (100)	32 (78.0)	9 (22.0)	41 (100)	16 (38.1)	26 (61.9)	42 (100)	32 (78.0)	9 (22.0)	41 (100)	22 (44.0)	28 (56.0)	50 (100)	14 (28.0)	36 (72.0)	50 (100)
**GR and PR dominant**	29 (23.4)	95 (76.6)	124 (100)	61 (49.2)	63 (50.8)	124 (100)	104 (90.4)	11 (9.6)	115 (100)	94 (76.4)	29 (23.6)	123 (100)	78 (61.4)	49 (38.6)	127 (100)	45 (38.1)	73 (61.9)	118 (100)	84 (70.6)	35 (29.4)	119 (100)	64 (53.8)	55 (46.2)	119 (100)

[a]cut-off value of **CD 4+** is over 0.19 of IHC score; [b]cut-off value of CD 8+ is over 1.33 of IHC score; [c]cut-off value of CD 4+/CD 8+ is over 4.73 of IHC score; [d]cut-off value of CD 3+ is over 24.53 of IHC score; [e]cut-off value of FoxP 3+ is over 0.36 of IHC score; [f]cut-off value of CD 3+/FoxP 3+ is over 23.21 of IHC score FIGO, [g]cut-off value of PD-1 is over 1.2; [h]cut-off value of PD-L1 is 1.6; International Federation of Gynecology and Obstetrics
