# Supplementary material for: Clinical Significance of Tumor Infiltrating Lymphocytes in Association with Hormone Receptor Expression Patterns in Epithelial Ovarian Cancer
Source: Int J Mol Sci. 2021 May 27;22(11):5714. doi: 10.3390/ijms22115714 (PMC8198528; doi:10.3390/ijms22115714)
Supplement: Supplementary file 1 [file ijms-22-05714-s001.zip › 5. Revision_Supplementary Table S5. Primary antibodies used in this study .pdf]

Supplementary Table S5. Primary antibodies used in this study

Antibody	Raised in	Clone/ Catalog No.	Dilution	Source[a]
ERα	mouse	ER-6F11-L-CE	1:300	Leica
AR	rabbit	760-4605	1:300	Roche
GR	rabbit	3660S	1:300	Cell Signaling Technology
PR	rabbit	NCL-L-PGR-312	1:300	Leica
ERβ	rabbit polyclonal	PU385-UP	1:300	BioGenex
$CD3^+$	rabbit polyclonal	Ab5690	1:300	Abcam
$CD4^+$	rabbit	EPR6855	1:100	Abcam
$CD8^+$	rabbit polyclonal	ab4500	1:200	Abcam
FoxP 3^+	mouse	14-4777-82	1:300	eBioscience
PD-1	mouse	315M-95	1:100	CellMarque
PD-L1	mouse	M365329-2	1:100	Dako

[a] Leica, Buffalo Grove, IL; Roche, Rocklin, CA; Cell Signaling Technology, Danvers, MA; BioGenex, Fremont, CA; Abcam, Cambridge, MA; eBioscience, San Diego, CA; CellMarque, Rocklin, CA; DAKO, Carpinteria, CA
