# Supplementary material for: Clinical Significance of Tumor Infiltrating Lymphocytes in Association with Hormone Receptor Expression Patterns in Epithelial Ovarian Cancer
Source: Int J Mol Sci. 2021 May 27;22(11):5714. doi: 10.3390/ijms22115714 (PMC8198528; doi:10.3390/ijms22115714)
Supplement: Supplementary file 1 [file ijms-22-05714-s001.zip › 6. Revision_Supplementary Figure S1.pdf]

A

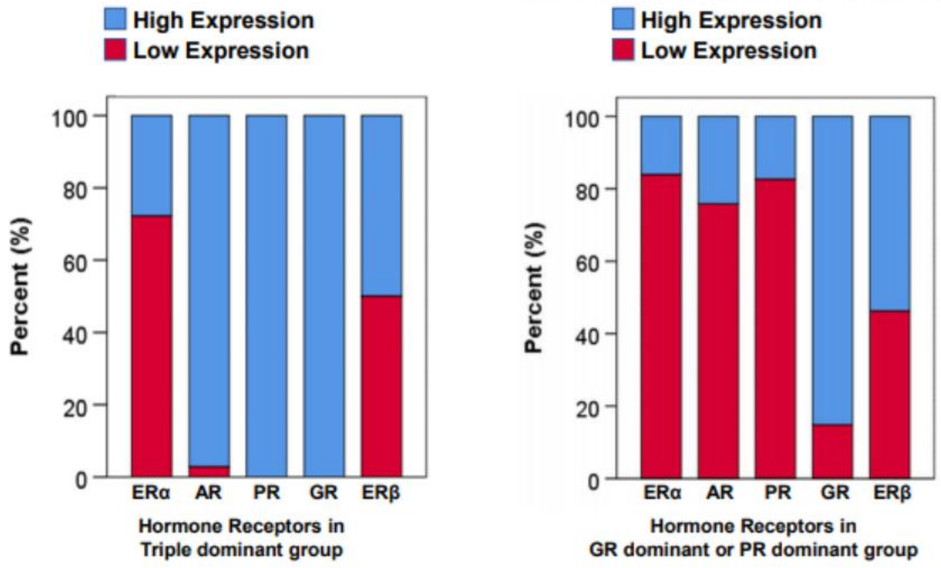

B

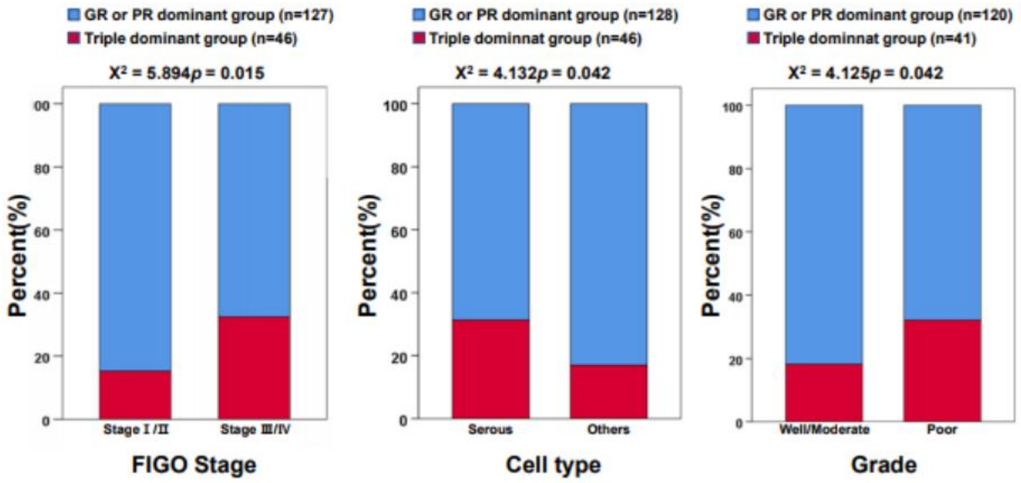

Supplementary Figure 1

Supplementary Figure S1. Characteristics of EOC subgroups clustered by hormone receptor expression pattern (A) Expression patterns of hormone receptors in the triple dominant group and GR- or PR-dominant group. (B) Clinicopathological analysis of the triple dominant group
