# Supplementary material for: Clinical Significance of Tumor Infiltrating Lymphocytes in Association with Hormone Receptor Expression Patterns in Epithelial Ovarian Cancer
Source: Int J Mol Sci. 2021 May 27;22(11):5714. doi: 10.3390/ijms22115714 (PMC8198528; doi:10.3390/ijms22115714)
Supplement: Supplementary file 1 [file ijms-22-05714-s001.zip › 7. Revision_Supplementary Figure S2.pdf]

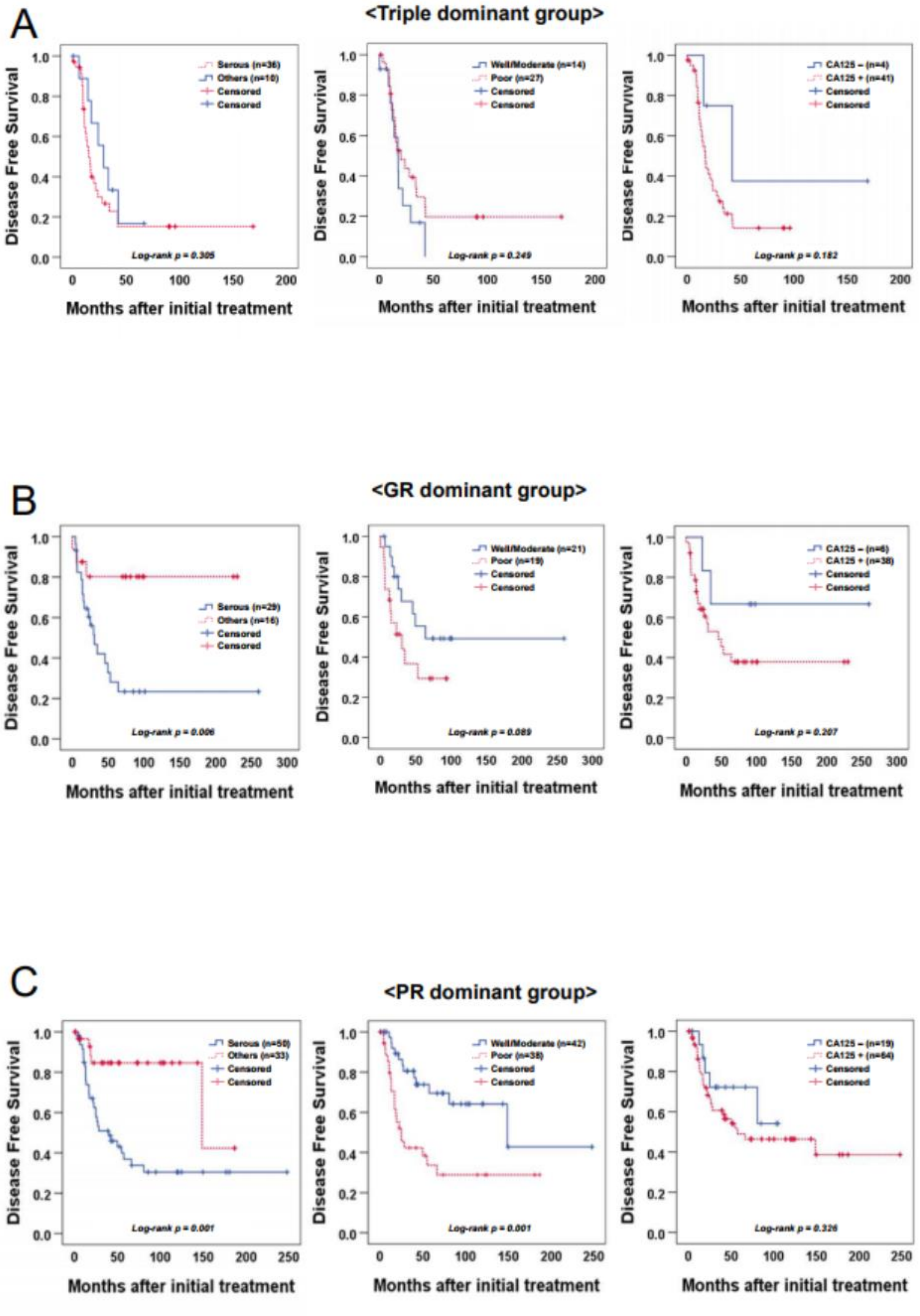

Supplementary Figure 2

Supplementary Figure S2. Subgroup disease free survival (DFS) analysis in the triple dominant, GR-dominant, and PR-dominant EOC groups. (A) DFS analysis in the triple dominant group based on cell type, grade, and CA125. (B) DFS analysis in the GR-dominant group based on cell type, grade, and CA125. (C) DFS analysis in the PR dominant group based on cell type, grade, and CA125.
