# Supplementary material for: Clinical Significance of Tumor Infiltrating Lymphocytes in Association with Hormone Receptor Expression Patterns in Epithelial Ovarian Cancer
Source: Int J Mol Sci. 2021 May 27;22(11):5714. doi: 10.3390/ijms22115714 (PMC8198528; doi:10.3390/ijms22115714)
Supplement: Supplementary file 1 [file ijms-22-05714-s001.zip › 8. Revision_Supplementary Figure S3.pdf]

<Triple dominant group>

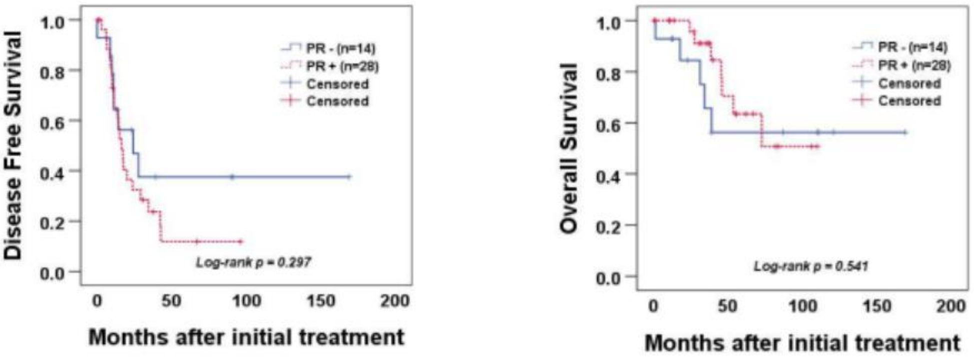

<GR dominant group>

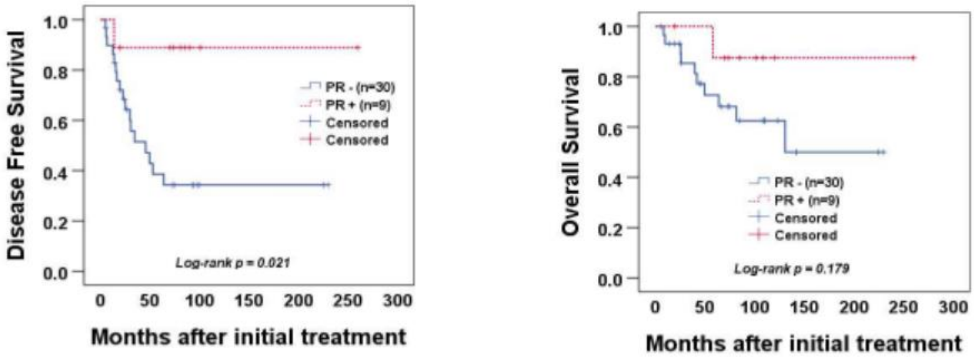

<PR dominant group>

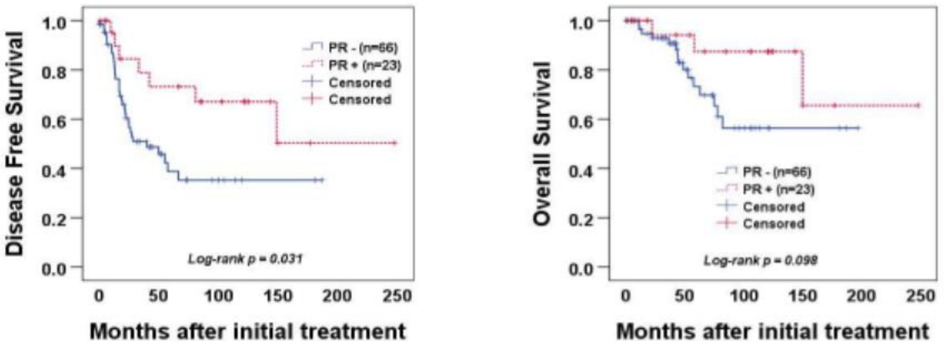

Supplementary Figure 3

Supplementary Figure S3. **Disease free survival (DFS) and overall survival (OS) analyses of the triple dominant, GR-dominant, and PR-dominant group depends on PR expression.**
