# Supplementary material for: Clinical Significance of Tumor Infiltrating Lymphocytes in Association with Hormone Receptor Expression Patterns in Epithelial Ovarian Cancer
Source: Int J Mol Sci. 2021 May 27;22(11):5714. doi: 10.3390/ijms22115714 (PMC8198528; doi:10.3390/ijms22115714)
Supplement: Supplementary file 1 [file ijms-22-05714-s001.zip › Supplementary_total.pdf]

Supplementary Table 1. Primary antibodies used in this study

Antibody	Raised in	Clone/ Catalog No.	Dilution	Source[a]
ERα	mouse	ER-6F11-L-CE	1:300	Leica
AR	rabbit	760-4605	1:300	Roche
GR	rabbit	3660S	1:300	Cell Signaling Technology
PR	rabbit	NCL-L-PGR-312	1:300	Leica
ERβ	rabbit polyclonal	PU385-UP	1:300	BioGenex
$CD3^+$	rabbit polyclonal	Ab5690	1:300	Abcam
$CD4^+$	rabbit	EPR6855	1:100	Abcam
$CD8^+$	rabbit polyclonal	ab4500	1:200	Abcam
FoxP 3^+	mouse	14-4777-82	1:300	eBioscience
PD-1	mouse	315M-95	1:100	CellMarque
PD-L1	mouse	M365329-2	1:100	Dako

[a] Leica, Buffalo Grove, IL; Roche, Rocklin, CA; Cell Signaling Technology, Danvers, MA; BioGenex, Fremont, CA; Abcam, Cambridge, MA; eBioscience, San Diego, CA; CellMarque, Rocklin, CA; DAKO, Carpinteria, CA

Supplementary Table S2. Clinico pathological characteristics of hormone receptors in epithelial ovarian cancer.

	ERα[a]			AR[b]			PR[c]			GR[d]			ERβ[e]		
	Low n (%)	High n (%)	Total n (%)	Low n (%)	High n (%)	Total n (%)	Low n (%)	High n (%)	Total n (%)	Low n (%)	High n (%)	Total n (%)	Low n (%)	High n (%)	Total n (%)
**Diagnosis**	*p < 0.001*			*p < 0.001*			*p < 0.001*			*p < 0.001*			*p = 0.060*		
**Normal**	75(94.9)	4 (5.1)	79(100)	56(70.9)	23 (29.1)	79(100)	36(46.2)	42(53.8)	78(100)	39(49.4)	40(50.6)	79(100)	33(41.8)	46(58.2)	79(100)
**Benign**	104(94.5)	6(5.5)	110(100)	83(74.8)	28(25.2)	111(100)	53(47.3)	59(52.7)	112(100)	14(11.4)	109(88.6)	123(100)	60(55.6)	48(44.4)	108(100)
**Borderline**	47(83.9)	9(16.1)	56(100)	32(58.2)	23(41.8)	55(100)	37(68.5)	17(31.5)	54(100)	1(1.8)	56(98.2)	57(100)	19(34.5)	36(65.5)	55(100)
**Cancer**	166(80.2)	41(19.8)	207(100)	76(39.6)	116(60.4)	192(100)	153(73.6)	55(26.4)	208(100)	21(10.0)	188(90.0)	209(100)	96(46.8)	109(53.2)	205(100)
**FIGO stage**	*p = 0.134*			*p = 0.092*			*p = 0.953*			*p = 0.050*			*p = 0.047*		
**I-II**	52(86.7)	8(13.3)	60(100)	19(33.9)	37(66.1)	58(100)	43(71.7)	17(28.3)	60(100)	11(18.3)	49(81.7)	60(100)	21(35.6)	38(64.4)	59(100)
**III-IV**	99(77.3)	29(22.7)	128(100)	54(45.8)	64(54.2)	118(100)	91(71.1)	37(28.9)	128(100)	11(8.5)	118(91.5)	129(100)	72(56.7)	55(43.3)	127(100)
**Cell type**	*p = 0.027*			*p = 0.126*			*p = 0.008*			*p = 0.391*			*p = 0.682*		
**Serous**	165(81.3)	38(18.7)	203(100)	111(56.9)	84(43.1)	195(100)	120(59.1)	83(40.9)	203(100)	13(6.5)	187(93.5)	200(100)	93(46.7)	106(53.3)	199(100)
**Others**	153(89.5)	18(10.5)	171(100)	81(49.4)	83(50.6)	164(100)	123(72.4)	47(27.6)	170(100)	15(8.9)	154(91.1)	169(100)	81(47.9)	88(52.1)	169(100)
**Tumor grade**	*p = 0.580*			*p = 0.080*			*p = 0.602*			*p = 0.090*			*p = 0.382*		
**Well/Moderate**	73(82.0)	16(18.0)	89(100)	29(34.1)	56(65.9)	85(100)	64(71.9)	25(28.1)	89(100)	15(16.9)	74(83.1)	89(100)	38(42.7)	51(57.3)	89(100)
**Poor**	82(78.8)	22(21.2)	104(100)	41(43.6)	53(56.4)	94(100)	96(75.2)	25(24.8)	101(100)	9(8.7)	94(91.3)	103(100)	50(49.0)	52(51.0)	102(100)
**CA125**	*p = 0.203*			*p = 0.024*			*p = 0.339*			*p = 0.787*			*p = 0.666*		
**Negative**	102(88.7)	13(11.3)	115(100)	65(60.2)	43(39.8)	108(100)	74(64.9)	40(35.1)	114(100)	9(8.2)	101(91.8)	110(100)	53(47.3)	59(52.7)	112(100)
**Positive (>35U/ml)**	165(83.3)	33(16.7)	198(100)	87(46.5)	100(53.5)	187(100)	137(69.5)	60(30.5)	197(100)	18(9.1)	180(90.9)	198(100)	88(45.6)	105(54.4)	193(100)
**Chemosensitivity**	*p = 0.784*			*p = 0.850*			*p = 0.111*			*p = 0.401*			*p = 0.115*		
**Sensitive**	137(79.2)	36(20.8)	173(100)	64(40.3)	95(59.7)	170(100)	128(71.3)	49(28.7)	171(100)	21(12.2)	151(87.8)	172(100)	76(44.7)	94(52.3)	170(100)
**Resistant**	13(76.5)	4(23.5)	17(100)	7(43.8)	9(56.2)	16(100)	16(88.9)	2(11.1)	18(100)	1(5.6)	17(94.4)	18(100)	11(64.7)	6(35.3)	17(100)

[a]cut-off value of **ERα** is over 49.2 of IHC score; [b]cut-off value of AR is over 10.85 of IHC score; [c]cut-off value of PR is over 21.18 of IHC score; [d]cut-off value of GR is over 8.65 of IHC score; [e]cut-off value of ERβ is over 105.97 of IHC score; FIGO, International Federation of Gynecology and Obstetrics

Supplementary Table S3. Clinico pathological characteristics of tumor infiltrating lymphocytes. PD-1 and PD-L1 in epithelial ovarian cancer.

Characteristic	CD 4^{+a}			CD 8^{+b}			CD 4^{+}/CD 8^{+c}			CD 3^{+d}			FoxP 3^{+e}			CD 3^{+}/FoxP 3^{+f}			PD-1+ ^{g}			PD-L1+^{h}		
	Low	High	Total	Low	High	Total	Low	High	Total	Low	High	Total	Low	High	Total	Low	High	Total	Low	High	Total	Low	High	Total
	- (n)	+ (n)	n (%)	- (n)	+ (n)	n (%)	- (n)	+ (n)	n (%)	- (n)	+ (n)	n (%)	- (n)	+ (n)	n (%)	- (n)	+ (n)	n (%)	- (n)	+ (n)	n (%)	- (n)	+ (n)	n (%)
**FIGO stage**	*p* = 0.021			*p* = 0.051			*p* = 0.071			*p* = 0.033			*p < 0.001*			*p* = 0.621			*p* = 0.016			*p* = 0.018		
**I-II**	19 (32.2)	40 (67.8)	59 (100)	33 (55.9)	26 (44.1)	59 (100)	52 (96.3)	2 (3.7)	54 (100)	50 (86.2)	8 (13.8)	58 (100)	45 (75.0)	15 (25.0)	60 (100)	26 (46.4)	30 (53.6)	56 (100)	45 (75.0)	15 (25.0)	60 (100)	36 (60.0)	24 (40.0)	60 (100)
**III-IV**	22 (17.2)	106 (82.8)	128 (100)	52 (40.6)	76 (59.4)	128 (100)	105 (87.5)	15 (12.5)	120 (100)	92 (71.9)	36 (28.1)	128 (100)	54 (42.5)	73 (57.5)	127 (100)	63 (46.4)	62 (49.6)	125 (100)	74 (56.1)	58 (43.9)	132 (100)	55 (41.7)	77 (58.3)	132 (100)
**Cell type**	*p* = 0.352			*p* = 0.017			*p* = 0.431			*p* = 0.049			*p* < 0.001			*p* = 0.794			*p* = 0.068			*p* = 0.211		
**Serous**	25 (19.8)	101 (80.2)	126 (100)	50 (39.7)	76 (60.3)	126 (100)	106 (89.1)	13 (10.9)	119 (100)	91 (72.2)	35 (27.8)	126 (100)	53 (42.4)	72 (57.6)	125 (100)	60 (48.8)	63 (51.2)	123 (100)	80 (55.9)	63 (44.1)	143 (100)	63 (43.8)	81 (56.3)	144 (100)
**Others**	16 (25.8)	46 (74.2)	62 (100)	36 (58.1)	26 (41.9)	62 (100)	52 (92.9)	4 (7.1)	56 (100)	52 (85.2)	9 (14.8)	61 (100)	47 (53.2)	16 (25.4)	63 (100)	30 (50.8)	29 (49.2)	59 (100)	47 (69.1)	21 (30.9)	68 (100)	36 (52.9)	32 (47.1)	68 (100)
**Tumor grade**	*p* = 0.210			*p* = 0.099			*p* = 0.811			*p* = 0.017			*p* = 0.119			*p* = 0.244			*p* = 0.018			*p* = 0.040		
**Well/Moderate**	21 (25.9)	60 (74.1)	81 (100)	43 (53.1)	38 (46.9)	81 (100)	67 (89.3)	8 (10.7)	75 (100)	68 (84.0)	13 (16.0)	81 (100)	48 (58.5)	34 (41.5)	82 (100)	35 (45.5)	42 (54.5)	77 (100)	60 (68.2)	28 (31.8)	88 (100)	47 (52.8)	42 (47.2)	89 (100)
**Poor**	16 (18.0)	73 (82.0)	89 (100)	36 (40.4)	53 (59.6)	89 (100)	76 (90.5)	8 (9.5)	84 (100)	60 (68.2)	28 (31.8)	88 (100)	41 (46.6)	47 (53.4)	88 (100)	48 (54.5)	40 (45.5)	88 (100)	54 (51.4)	51 (48.6)	105 (100)	40 (38.1)	65 (61.9)	105 (100)
**CA125**	*p* < 0.001			*p* = 0.012			*p* = 0.286			*p* = 0.477			*p* = 0.285			*p* = 0.042			*p* = 0.561			*p* = 0.290		
**Negative**	15 (53.6)	13 (46.4)	28 (100)	19 (67.9)	9 (32.1)	28 (100)	24 (96.0)	1 (4.0)	25 (100)	22 (81.5)	5 (18.5)	27 (100)	18 (62.1)	11 (37.9)	29 (100)	18 (66.7)	9 (33.3)	27 (100)	23 (65.7)	12 (34.3)	35 (100)	19 (54.3)	16 (45.7)	35 (100)
**Positive (>35 U/mL)**	26 (16.6)	131 (83.4)	157 (100)	66 (42.0)	91 (58.0)	157 (100)	131 (89.1)	16 (10.9)	147 (100)	118 (75.2)	39 (24.8)	157 (100)	80 (51.3)	76 (48.7)	156 (100)	69 (45.4)	83 (54.6)	152 (100)	104 (60.5)	68 (39.5)	172 (100)	77 (44.5)	96 (55.5)	173 (100)
**Chemosensitivity**	*p* = 0.216			*p* = 0.287			*p* = 0.635			*p* = 0.758			*p* = 0.375			*p* = 0.576			*p* = 0.355			*p* = 0.594		
**Sensitive**	33 (20.9)	125 (79.1)	128 (100)	67 (42.4)	91 (57.6)	158 (100)	129 (89.0)	16 (11.0)	145 (100)	118 (75.2)	39 (24.8)	157 (100)	77 (48.7)	81 (51.3)	158 (100)	75 (49.3)	77 (50.7)	152 (100)	104 (59.1)	72 (40.9)	176 (100)	80 (45.5)	96 (54.5)	176 (100)
**Resistant**	1 (7.1)	13 (92.9)	14 (100)	8 (57.1)	6 (42.9)	14 (100)	13 (92.9)	1 (7.1)	14 (100)	10 (71.4)	4 (28.6)	14 (100)	8 (61.5)	5 (38.5)	13 (100)	8 (57.1)	6 (42.9)	14 (100)	12 (70.6)	5 (29.4)	17 (100)	7 (38.9)	11 (61.1)	18 (100)
**Hormone receptor**	*p* = 0.656			*p* = 0.258			*p* = 0.389			*p* = 0.831			*p* = 0.008			*p* < 0.001			*p* = 0.001			*p* = 0.002		
**Triple dominant**	11 (26.8)	30 (73.2)	41 (100)	16 (39.0)	25 (61.0)	41 (100)	37 (94.9)	2 (5.1)	39 (100)	32 (78.0)	9 (22.0)	41 (100)	16 (38.1)	26 (61.9)	42 (100)	32 (78.0)	9 (22.0)	41 (100)	22 (44.0)	28 (56.0)	50 (100)	14 (28.0)	36 (72.0)	50 (100)
**GR and PR dominant**	29 (23.4)	95 (76.6)	124 (100)	61 (49.2)	63 (50.8)	124 (100)	104 (90.4)	11 (9.6)	115 (100)	94 (76.4)	29 (23.6)	123 (100)	78 (61.4)	49 (38.6)	127 (100)	45 (38.1)	73 (61.9)	118 (100)	84 (70.6)	35 (29.4)	119 (100)	64 (53.8)	55 (46.2)	119 (100)

[a]cut-off value of **CD 4+** is over 0.19 of IHC score; [b]cut-off value of CD 8+ is over 1.33 of IHC score; [c]cut-off value of CD 4+/CD 8+ is over 4.73 of IHC score; [d]cut-off value of CD 3+ is over 24.53 of IHC score; [e]cut-off value of FoxP 3+ is over 0.36 of IHC score; [f]cut-off value of CD 3+/FoxP 3+ is over 23.21 of IHC score FIGO, [g]cut-off value of PD-1 is over 1.2; [h]cut-off value of PD-L1 is 1.6; International Federation of Gynecology and Obstetrics

Supplementary Figure S1. Characteristics of EOC subgroups clustered by hormone receptor expression pattern (A) Expression patterns of hormone receptors in the triple dominant group and GR- or PR-dominant group. (B) Clinicopathological analysis of the triple dominant group

A

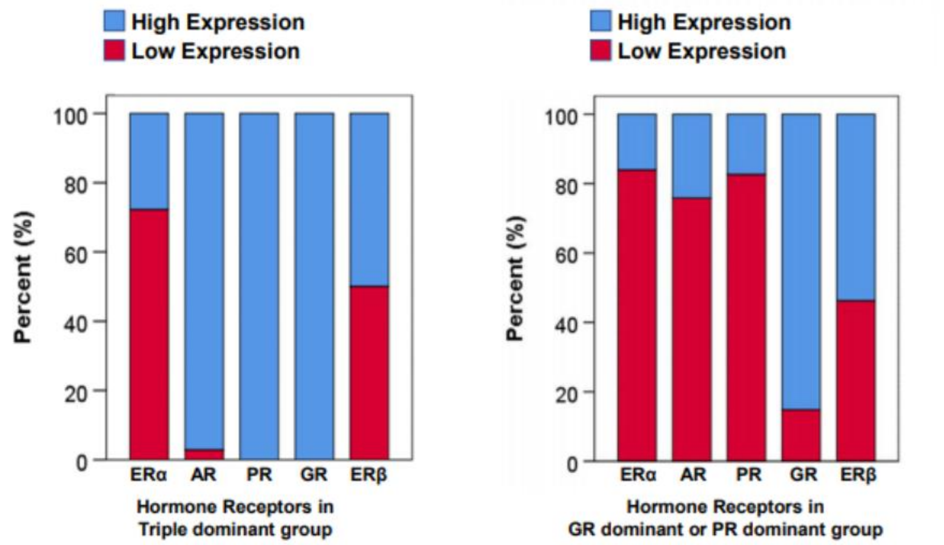

B

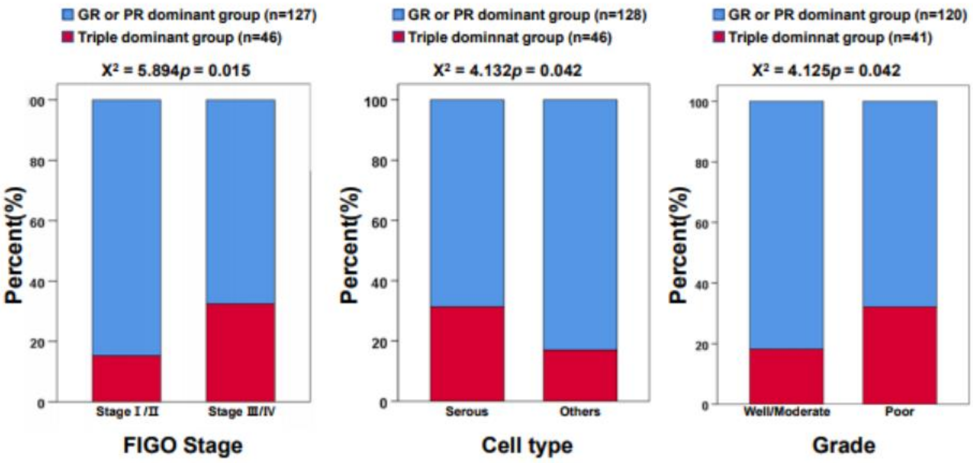

Supplementary Figure 1

Supplementary Figure S2. Subgroup disease free survival (DFS) analysis in the triple dominant, GR-dominant, and PR-dominant EOC groups. (A) DFS analysis in the triple dominant group based on cell type, grade, and CA125. (B) DFS analysis in the GR-dominant group based on cell type, grade, and CA125. (C) DFS analysis in the PR dominant group based on cell type, grade, and CA125.

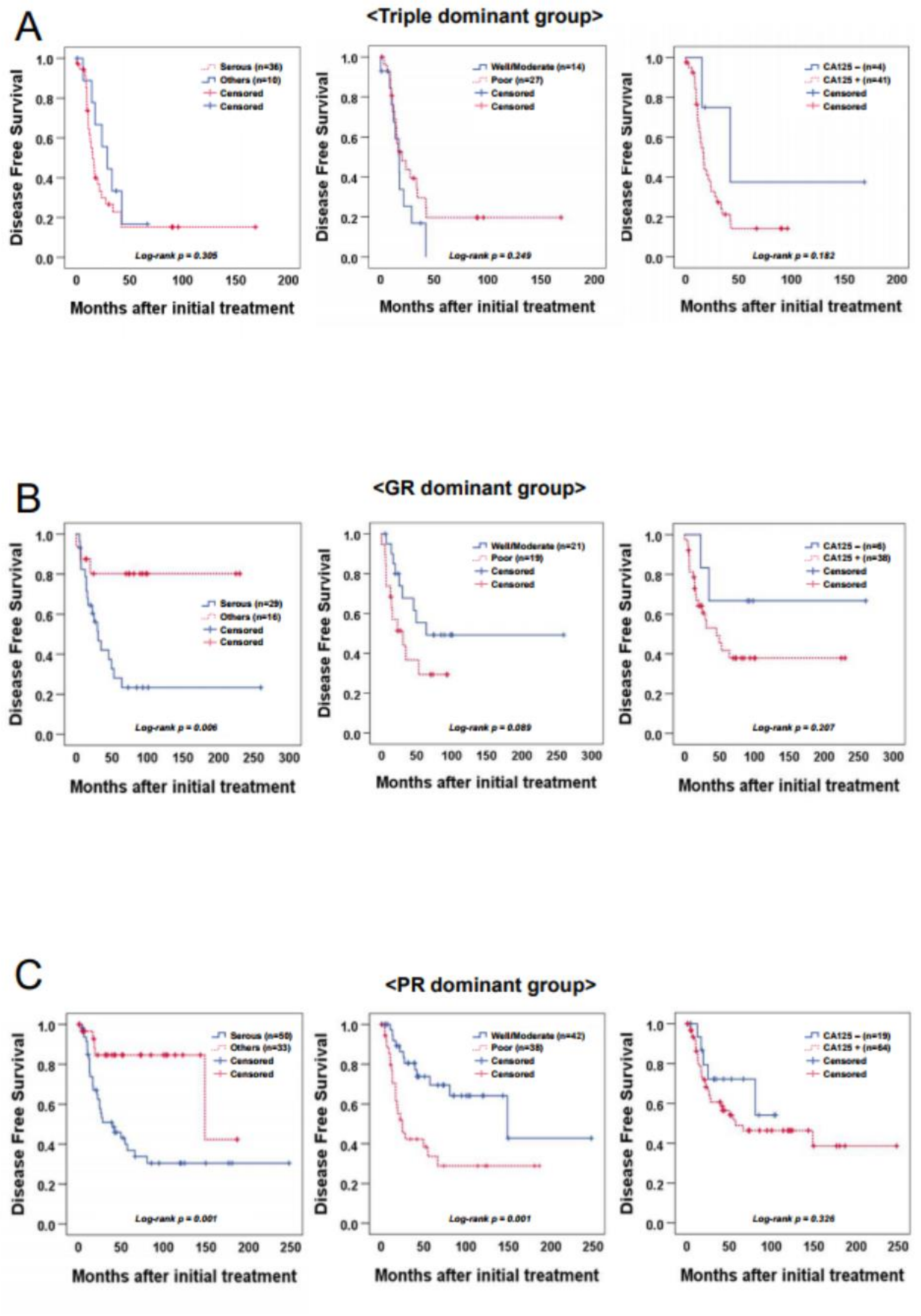

Supplementary Figure 2

Supplementary Figure S3. **Disease free survival (DFS) and overall survival (OS) analyses of the triple dominant, GR-dominant, and PR-dominant group depends on PR expression.**

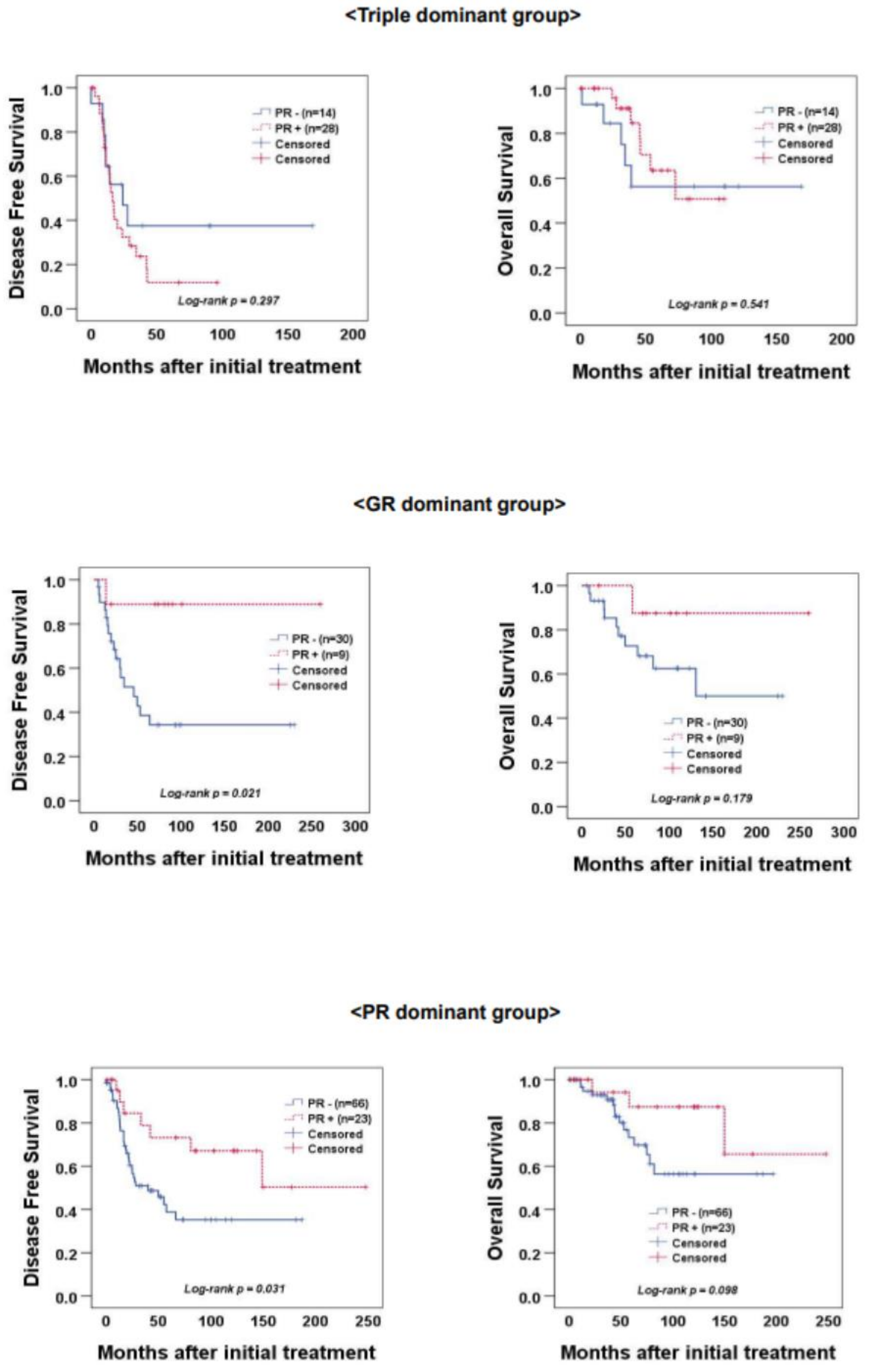

Supplementary Figure 3
